# Colourful agrobiodiversity: morphology and phenology of bean landraces to face commodification of the commons in the southern Andes

**DOI:** 10.1186/s40529-025-00488-6

**Published:** 2026-01-15

**Authors:** María J. Romero-Silva, Rayén Liechti-Araneda, J. Tomás Ibarra-Eliessetch, Constanza Monterrubio-Solís, Antonia Barreau-Daly

**Affiliations:** 1 Porotarium Austral SpA, Pucón, La Araucanía Region Chile; 2 Center for Local Development (CEDEL) & Center for Intercultural and Indigenous Research (CIIR), ECOS (Ecosystem-Complexity-Society) Co-Laboratory, Pontificia Universidad Católica de Chile, Villarrica Campus, Araucanía, Chile; 3 Department of Ecosystems and Environment, Faculty of Agriculture and Natural Systems, Center of Applied Ecology and Sustainability (CAPES), Pontificia Universidad Católica de Chile, Santiago, Chile; 4 Cape Horn International Center for Global Change Studies and Biocultural Conservation (CHIC), Universidad de Magallanes, Puerto Williams, Chile; 5 Department of Political and Social Sciences, Johns Hopkins University-Universitat Pompeu Fabra Public Policy Center (JHU-UPF PPC), Barcelona, Spain

**Keywords:** *Phaseolus*, Beans, Landraces, Varieties, Local, Agrobiodiversity, Biocultural conservation

## Abstract

In recent decades, agrobiodiversity has significantly declined due to genetic erosion. Conservation strategies in response include both ‘in situ’ and ‘ex situ’ methods, with a third approach called ‘in vivo’ conservation, standing out as a promising approach for conserving agrobiodiversity within active agricultural systems by maintaining live cultivars rather than storing frozen genetic material. Bean landraces (*Phaseolus* spp.) from southern South America have been cultivated for more than a thousand years, and thus hold deep cultural and ecological significance; however, little information exists about their morphological and phenological characteristics. We propagated bean landraces in homegardens over two agricultural seasons (2022–2024) in the La Araucanía region, southern Chile. We implemented a transdisciplinary and participatory approach involving local women gardeners, with whom we conducted in vivo conservation trials in homegardens, under real cultivation conditions, integrating botanical characterization with traditional knowledge and local management practices. This approach allowed the documentation, for the first time, of phenological and morphological diversity of 30 bean landraces while strengthening community-based conservation. Detailed phenological and agrobotanical traits were recorded, including growth habits, morphological characteristics, and seed metrics. The collected data were analyzed using Principal Component Analysis to identify patterns and correlations among traits. We found significant morphological variability, especially in leaf, flower, pod, and seed traits, highlighting the potential to identify subgroups within the landraces. This work not only supports the conservation of these valuable landraces but also strengthens the local food systems, offering a pathway for communities to face commodification of this agrobiodiversity in order to safeguard their agricultural heritage and enhance local social-ecological resilience.

**Supplementary Information:**

The online version contains supplementary material available at 10.1186/s40529-025-00488-6.

## Background

The term agrobiodiversity refers to the diversity and variability of organisms that contribute to human nutrition, associated with agriculture and livestock in complex ecological systems (Jackson et al. 2013; Qualset et al. 1995). Over the past decades, the diversity of plants in the diets of numerous human societies has rapidly declined (Ickowitz et al. 2019; Khoury et al. 2014), as evidenced by genetic erosion (Van de Wouw et al. 2010), a process which results in the loss of both individual genes and their combinations, including entire crop varieties. Its primary cause is modern high-yielding varieties replacing local landraces, which are traditional varieties of ancestral crops characterized by their adaptation to the edaphoclimatic conditions of local territories and by their strong cultural and historical ties to the regions from which they originate (Bocci and Chable 2009; Kastler et al. 2013; Santillán 2018; Ibarra et al. 2024b). Genetic erosion accelerated during the Green Revolution in the 1950s and gained renewed momentum in the new millennium with the emergence of genetically modified organisms (Ceccon 2008). Other contributing factors include human activities such as ecosystem overexploitation, overgrazing, urban pressures, and shifts in human diets (Qualset et al. 1997; Thormann and Engels 2015).

Losing agrobiodiversity creates vulnerability because a widely distributed and intensively managed crop is uniformly susceptible to a hazard posed by a pest, pathogen, or environmental factor due to its genetic composition, thereby opening the possibility of widespread crop losses (Visser et al. 2009). Therefore, conserving agrobiodiversity, especially landraces, is essential for addressing challenges such as climate change and disease adaptation, strengthening both food sovereignty and security (Mishra et al., 2024; Tscharntke et al. 2012; Visser et al. 2009). To achieve this, various approaches have emerged over the past decades. On the one hand, there are seed banks, which aim to conserve crop genetic material ex situ by freezing and storing it for long periods at relatively low cost (Diulgheroff and Robinson 2009; Li and Pritchard 2009; Peres 2016; Vernooy et al. 2016). There are over 1,750 plant gene banks worldwide, with the largest including the International Maize and Wheat Improvement Center, the International Rice Research Institute, Plant Genetic Resources Canada, and the International Center for Tropical Agriculture in Colombia, which is notable for housing the largest collection of bean (*Phaseolus* spp.) germplasm (Diulgheroff and Robinson 2009). Additionally, the Svalbard Global Seed Vault serves as a backup repository for other seed banks (Börner and Khlestkina 2019). However, these banks are also susceptible to genetic erosion due to, for example, the methods used to collect their samples (Gómez-Campo 2006; Rogers 2004).

On the other hand, there is in situ conservation, which involves the conservation and protection of biodiversity in its natural habitat and, in the case of cultivated species, in the locations where their specific traits have developed (Dulloo et al. 2009). This is considered one of the most effective strategies to combat genetic erosion (Visser et al. 2009). Today, a variant called in vivo conservation has emerged, and it entails safeguarding and reproducing species and/or varieties of interest for current and future generations within the everyday practices of cultivation and their associated biocultural derivatives (Nazarea 2006). The latter highlights the role of farmers as the actors who actively maintain agrobiodiversity, instead of storing them away in seed banks. Landraces persist over time thanks to small-scale farmers who traditionally have cultivated, harvested, classified, conserved, reproduced, and shared landrace seeds and propagules with family members and other social actors in local territories (Ibarra et al. 2019). Landraces are generally managed using traditional techniques, without the use of commercial agrochemicals (Red de Guardianes de Semillas 2024). In this way, landraces embody a set of ecological, sociocultural, political, and religious-spiritual values and form a central part of the commons of their territories (Kastler et al. 2013; Ibarra et al. 2023; Rodríguez 2023). These commons constitute essential collective goods for life, culture, and history, which are produced, transmitted, and inherited within communities and peoples (Espeleta and Moraga 2011; Perelmuter 2014). Due to their unique traits and their connection to communities, landraces can only be conserved through their cultivation (Santillán 2018). This involves working in homegardens, which are small-scale farming systems that integrate food production, agrobiodiversity conservation, and cultural practices, and are primarily developed by women (Barreau et al. 2019; Ibarra and Barreau 2019; Ibarra et al. 2019; Mies and Shiva 1998).

Legumes, including pulses, are a critically important botanical group for food security due to their high nutritional value as a key source of plant-based protein, their role in preventing non-communicable chronic diseases, and their high nitrogen-fixing capacity, which helps mitigate climate change and enhance social-ecological resilience (FAO, 2024; Kushi et al. 1999; Nyau 2014; Thompson 2019). Additionally, compared to meat as a protein source, pulses usually have lower lipid content, a reduced carbon footprint, and lower costs for both producers and consumers (Gazan et al. 2021; Havemeier et al. 2017; Thompson 2019). Despite these benefits, global consumption of pulses has decreased over the past 100 years, shifting from a staple food to an occasional one (Thompson 2019). As noted by Afshin et al. (2019) in a study spanning 195 countries, only regions such as the Caribbean, tropical Latin America, South Asia, and Eastern and Western Africa meet the recommended consumption levels, while in Western countries, pulse consumption remains low in non-vegetarian diets (Havemeier et al. 2017; Orlich et al. 2014). In response to this trend, various campaigns have emerged to promote pulse consumption all around the world, including the ‘International Year of Pulses’, which aimed to highlight their benefits (FAO, 2024), ‘Bean is How’, seeking to double global bean consumption by 2028 (Didinger et al. 2024), the 317 legume varieties registered in ‘Slow Food’s Ark of Taste’, whose mission is to share and safeguard their biodiversity (Milano et al. 2018), the ‘Pan-Africa Bean Research Alliance’, focused on improving food security and health (Pan Africa Bean Research Alliance 2022), the ‘Global Bean Project’, aiming to increase the cultivation and consumption of legumes around the world (Global Bean Project 2025), the ‘Legume Hub Community’, providing multi-lingual access to legume crop production knowledge (Watson and Murphy-Bokern 2022), and the ‘Beany Things’, focused on testing and conserving landraces in the European Union (Beany Things 2024).

Among legumes, beans (*Phaseolus* spp.), including species such as the common bean (*P. vulgaris*), scarlet runner bean (*P. coccineus*), lima bean (*P. lunatus*), year bean (*P. dumosus*), and tepary bean (*P. acutifolius*), are the most important for human consumption, accounting for half of global legume production (Broughton et al. 2003; Bitocchi et al. 2017). Latin America is the largest producer of these legumes (Cardona 2004), and beans are also a fundamental part of the biocultural heritage of southern South America (Pastor and Gil 2014), where they have been cultivated for over a thousand years (Campbell et al. 2018; Roa et al. 2015). Within Chile, the *Chile landrace* of beans has been recognized, identifying the country as a secondary domestication center for the beans in South America (Bascur and Tay 2005). In Chile, bean cultivation is deeply rooted in small-scale farming (Baginsky and Ramos 2018), which accounts for 70–80% of the national production. Culturally, beans are among the most valued seeds in small-scale farms, they are nurtured and passed down through generations of campesino families, and embody a key biocultural heritage (Ibarra et al. 2024a) due to their high nutritional value, versatility (consumed fresh, in pods, or dried), diversity (in size, color, and flavor), and the aesthetic appeal of the seeds themselves (Semilla Austral 2023). Despite their importance, as with other legumes, bean consumption is declining in southern South America (Granado, 2021), including Chile, alongside a reduction in the hectares allocated for their cultivation in the country (Baginsky and Ramos 2018; Pinheiro et al. 2018).

In response, the National School Assistance and Scholarship Board has promoted menus featuring legumes as a protein source for students, noting that “in the past, they were a staple of the traditional Chilean diet, which has been disappearing along with the benefits they provide” (JUNAEB 2024), while also emphasizing their cultural significance. In southern Chile, within the La Araucanía region, 60% of small-scale farming is carried out by Indigenous Mapuche families, based on their traditional practices (Biodiversidad Alimentaria & CONADI, 2020; Órdenes et al., 2023). Beans also hold a central place in Mapuche culinary traditions, being featured in daily and festive dishes such as *porotos granados*, and *porotos con mote*, which embody ancestral knowledge of nourishment, reciprocity, and care for the land. This biocultural dimension reinforces their symbolic and nutritional importance within food systems and underpins the relevance of conserving these landraces in vivo. As such, traditional knowledge regarding the agrobotanical traits, management practices, and consumption of landraces is crucial for their agricultural production and the conservation of their diversity (Grajales 2010). This protection is recognized as a right under the United Nations Declaration on the Rights of Peasants and Other People Working in Rural Areas (UNDROP 2018). However, as this knowledge is primarily transmitted orally and not formally documented, there is a significant risk of biocultural memory loss. This vulnerability is exacerbated by the impacts of the Green Revolution, which introduced shifts toward commercial crops and the use of techniques such as agrochemicals and agricultural machinery (Ceccon 2008). Consequently, the cultivation and consumption of these landraces are gradually disappearing, resulting in genetic erosion and a loss of agrobiodiversity in territories and communities (Biodiversidad Alimentaria & CONADI, 2020).

**Fig. 1:**
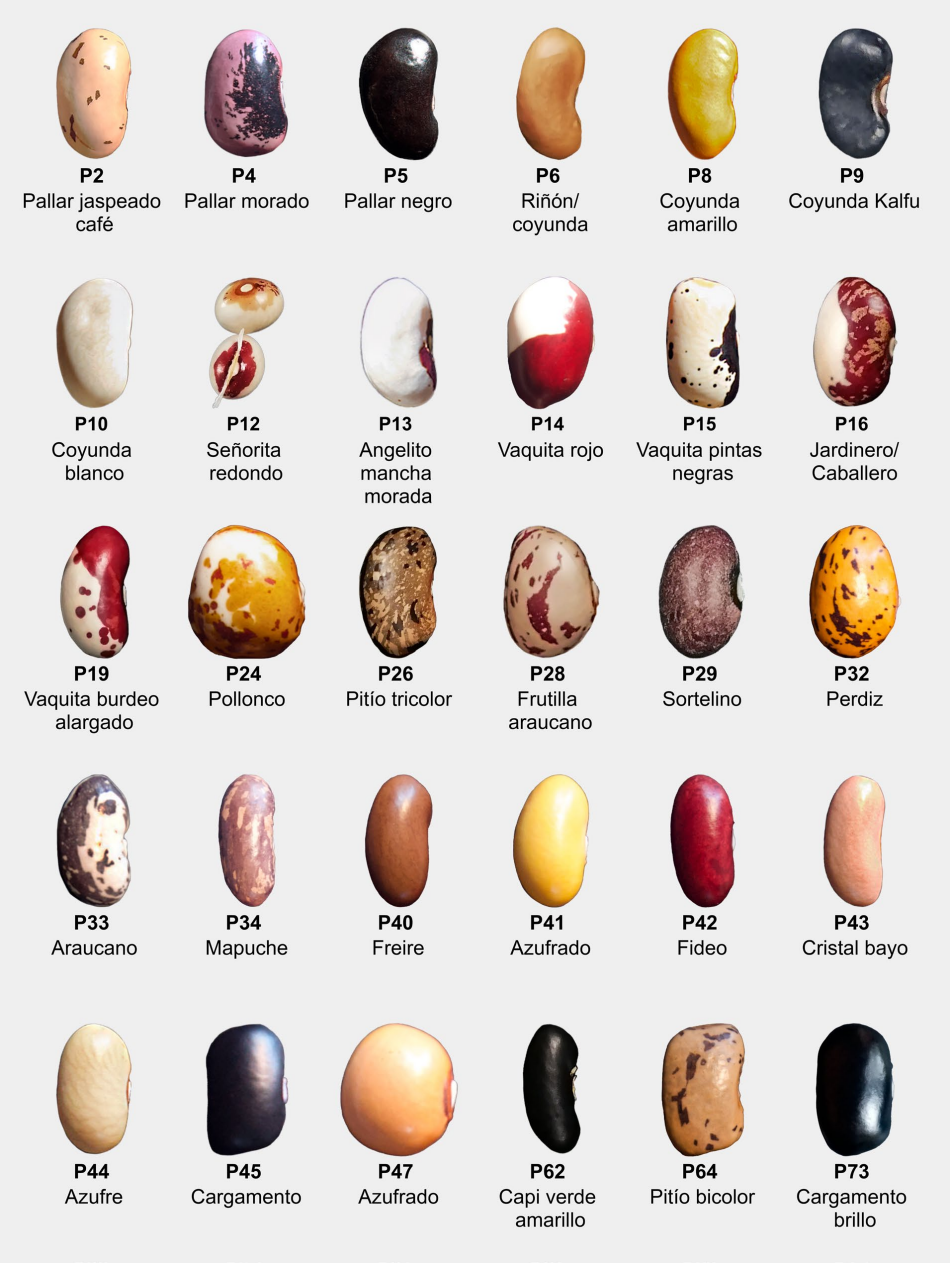
Traditional bean landraces cultivated during two agricultural seasons of Porotarium Austral, Araucanía Region, southern Chile. Seed pictures taken as photographic records from the Porotarium Austral Seed Reserve. PA internal code denotes order of addition to the bank. Common names obtained from local bean farmers

In this study, we document, describe, and compare the agrobotanical, morphological, and phenological traits of 30 bean landraces (Fig. 1) from the southern Andes in the La Araucanía region of Chile, within the context of the *Porotarium Austral Seed Refuge*, which houses more than 90 accessions. The documentation and analysis of their morphological and phenological traits will not only help prevent genetic erosion but also strengthen food sovereignty and the social-ecological resilience of communities. In the context of the looming threat of landraces being replaced by commercial varieties, this work is essential for safeguarding traditional knowledge and ensuring long-term food security (Akinola et al. 2020). Beans, and the landraces that constitute the agrobiodiversity of homegardens in southern Chile, are intrinsically linked to the territories and small-scale farming; they are common goods whose value transcends the economic, being fundamental for biodiversity, social-environmental justice, and the food autonomy of local communities. This effort could not only support the conservation of their genetic diversity but also enable communities to resist processes of commodification and privatization of common goods, which is essential for the livelihoods of small-scale farmers (Ibarra et al. 2023; Oduoye et al. 2024; Polanyi 2001). Our objective is to ensure that the registration and systematization of information about these landraces strengthens local in situ and in vivo conservation processes, thereby preventing genetic erosion but also commodification of our studied landraces. This approach aims to ensure that landraces remain an accessible and shared common good, vital for the autonomy, resilience, and food sovereignty of these territories.

## Methods

### Territorial cadastre and creation of Porotarium Austral Seed Refuge

In 2022, an initial cadastre was conducted through meetings with seed curators, gardeners, and vegetable growers from the municipalities of Pucón, Curarrehue, and Padre Las Casas, in the La Araucanía Region of Chile. Through semi-structured interviews, participants identified, from a selection of 66 landraces, those they knew and cultivated, noting their names and other information related to their cultivation and consumption. From these meetings, we established the Porotarium Austral Seed Refuge, a seed bank and repository of landrace knowledge, with the aim of conserving and promoting these landraces. The seed refuge is managed communally by the farmers and is updated with each agricultural season, in addition to new accessions that emerge from participation in seed exchanges (*Trafkintu*; Salazar et al., 2025). 

### In vivo propagation of bean landrace seeds

We propagated 30 bean landraces in vivo within family gardens over two study seasons, monitoring their development from sowing, through full plant growth, to seed harvest, documenting their phenology and agrobotanical traits of interest. All seeds were sourced from the Porotarium Austral Seed Refuge and were selected based on availability and cultural importance. Propagation took place between October and April of 2022–2023 (season 1) and 2023–2024 (season 2).

**Fig. 2:**
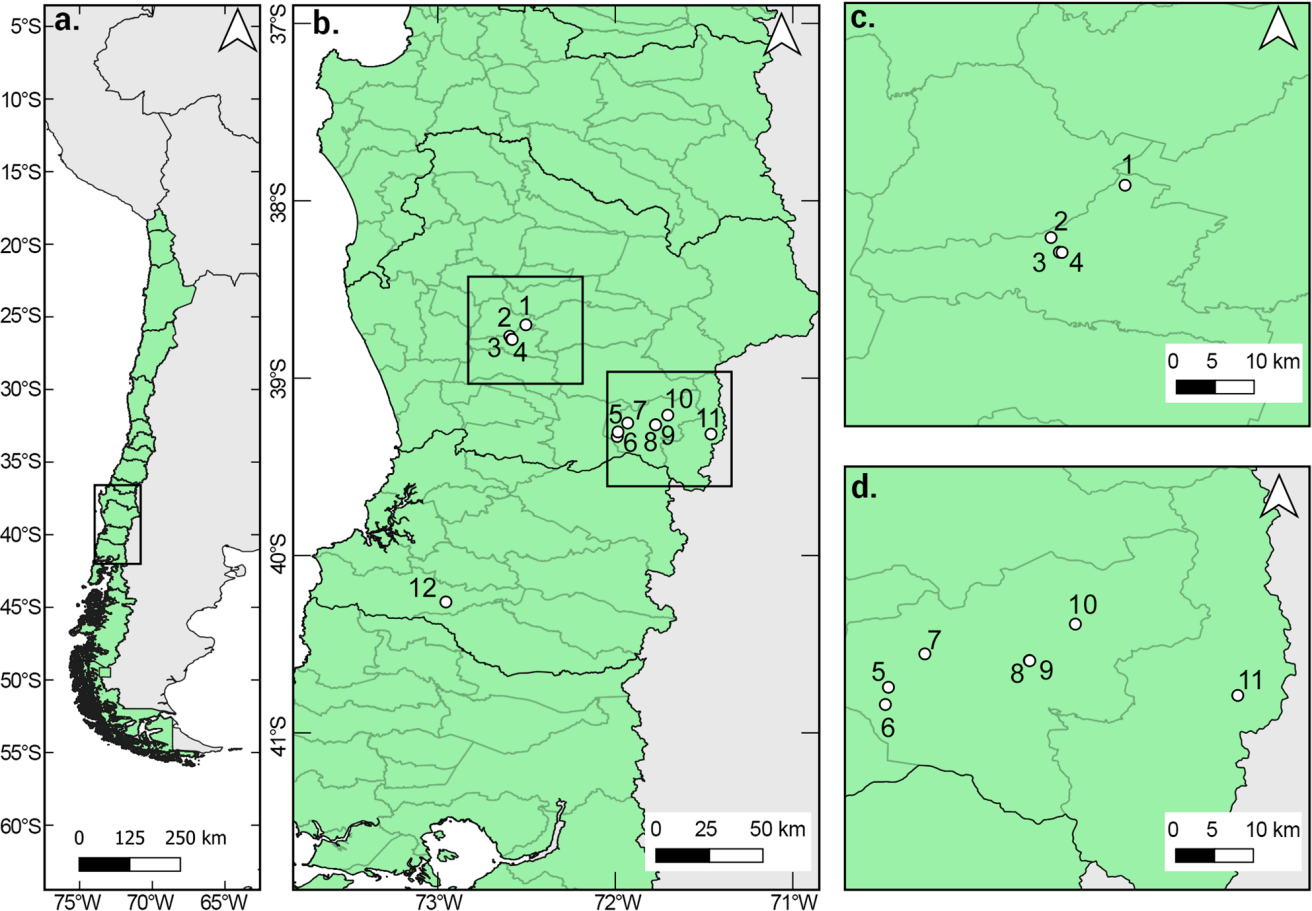
Geographical distribution map of the Porotarium Austral Seed Bank gardens. (**a**) General context of the study area. (**b**) Zoomed-in view of the study area showing the location of the gardens with numbered markers on the map. (**c**) Distribution of gardens in the Padre Las Casas municipality. (**d**) Distribution of gardens in the Pucón and Curarrehue municipalities

During the first season, sowing was conducted in nine homegardens located in the municipalities of Padre Las Casas, Pucón, and Curarrehue, each under the care of a female gardener. This emphasis on female gardeners acknowledges their central role in the transmission of traditional agricultural knowledge and in the conservation of local seed diversity in southern Chile (Peralta et al. 2013). Additionally, two gardens were managed under the care and supervision of the research team—one in La Unión, Los Ríos Region, and the other in Pucón, under co-supervision (Fig. 2). In this context, “co-supervision” refers to the shared management of the experimental garden between a researcher from the team and a local gardener, ensuring both scientific rigor and local experiential input.

Each gardener was responsible for four to seven landraces, and each landrace was cultivated in at least two different homegardens as a replicate. Each gardener received 50 seeds of each landrace, which were directly sown “three seeds at a time” (or two in case of seed shortage), with a distance of one foot between plants. The other planting aspects (soaking, arrangement, row spacing) and agricultural management practices (irrigation, weeding, hilling, amendments, treatments, associations) were at the discretion of each gardener, based on their customary practices and knowledge of their land conditions, and therefore heterogeneous. At the end of season 1, seeds were harvested and added to the Seed Refuge to be used in the next season and in seed exchanges. To prevent hybridization, we established a minimum distance between landraces, and separated those of the same species or with highly similar traits. Hybridization is an uncommon process, and it had a low occurrence between season 1 and 2. If hybridization was suspected, the seeds were removed from the Seed Refuge to protect the landraces. Although detailed edaphic and topographic parameters were not systematically recorded, the participating homegardens differed in altitude, slope, and soil type, which likely influenced the expression of morphological and phenological traits.

For the second agricultural season, we made adjustments to the sowing methodology based on the experience from season 1. Seeds for the second season were sourced from the same landrace batches and spatial isolation between varieties minimized cross-pollination. We identified the seedling growth stage as critical, as it is particularly vulnerable to frost damage, lack of irrigation, and wildlife attacks. Therefore, to reduce losses, seedlings were sown in nursery trays and transplanted in mid-November. Traditionally, according to local peasant knowledge and customs in southern Chile, beans are sown after November 1st, following the last “black frost” of spring. However, during the second season, it was collectively agreed with the gardeners to transplant in the second half of November due to frosts during the first half of the month that occurred in our first agricultural season (causing crop losses) and in the previous two years, which is evidence of climate change. At the time of transplanting, the seedlings had at least one pair of true leaves. The number of seeds was 144 (two trays of seedlings, 6 × 12) per landrace. In the second season, the study area was focused solely on the municipality of Pucón, and the number of gardens was reduced to six to enable closer and more frequent monitoring. We conducted monthly visits to the gardens to record the plants’ phenology in detail, particularly during the pod formation stage. Additionally, a registration protocol was established for digital recording and remote monitoring of the growth stages conducted by the gardeners.

### Trait measurement

We recorded morphological traits in at least two different gardens for each landrace seed during both agricultural seasons of the study. For each landrace, we described: (1) growth habit with the categories bush, half-runner, or pole, determined by the plant’s growth type (Debouck and Hidalgo 1985). (2) stem thickness, color, texture, and pubescence; (3) shape, color, thickness, texture, and pubescence of the leaf; (4) flower traits; (5) inflorescence traits; (6) color, pattern, and shape of the pod profile; (7) tip and curvature of the pod.

Within each landrace, we measured five individuals for: (1) total height, measured in centimeters from the ground to the apical tip; (2) maximum diameter, measured in centimeters at the widest transversal section of the plant; (3) internode distance, measured in centimeters between the nodes in the middle section of the plant; (4) number of nodes before the first flower, measured in units from the ground to the first node showing a reproductive bud. Within each of the five individuals, we also recorded the following three measurements: (1) length and width of the three leaflets (left, central, and right), measured in centimeters; (2) length of the leaf petiole and the petiolule of the central leaflet, measured in centimeters; (3) length of the pod measured in a straight line from the junction of the peduncle to the apex, in centimeters. We conducted the leaf measurements at the middle height of the plant, and the pod measurements on mature pods with formed beans (“granados”), before they dried.

After seed harvest, we recorded the width, length, and depth measurements of the seeds for each landrace using a caliper, and calculated the average seed volume by multiplying these three values. We also measured the 100 seed weight using a scale. The recording of phenological data (flowering times, pod development) was carried out through participatory research in collaboration with the gardeners. We introduced a data collection methodology, and based on this, they made continuous photographic records in the garden using their cell phone cameras, which complemented the records from the visits to the garden.

### Principal component analysis

We conducted a Principal Component Analysis (PCA), as suggested by Manly and Navarro (2016) to reduce a large number of related variables, such as morphoagronomic variables (Bria et al. 2019; Christina et al. 2021; Mendoza et al. 2006; Mohebodini et al. 2017; Vargas-Vázquez et al. 2008, 2011), across 15 traits for 30 bean landraces (Table 1, detailed in Appendix 5), with the aim of observing how non-quantitative traits relate to qualitative ones and identifying any patterns or correlations. We standardized the variables to have unit variance before the analyses, which were performed using R software (v4.3.2, R Core Team 2023) with the FactoMineR package for multivariate analysis (Lê et al. 2008).

**Table 1:** Qualitative and quantitative traits of 30 bean landraces

PA Code	Growth Habit	Height (cm)	Leaflet length (cm)	Petal color	Pod length (cm)	Pod color	Pod pattern color	Pod shape	Seed size ͥ	Seed shape	Seed primary color	Seed pattern
P2	Pole	177	8 ± 1	Red	12 ± 2	Yellow	Purple	Curved	Large	Kidney	Beige	Mottled
P4	Pole	276	7 ± 1	Red	11 ± 2	Yellow	Purple	Curved	Large	Kidney	Purple	Mottled
P5	Pole	350	9 ± 1	Red	14 ± 3	Yellow	Purple	Semi curved	Large	Kidney	Black	Solid
P6	Pole	280	10 ± 0	Purple	17 ± 2	Purple	Solid	Semi curved	Medium	Kidney	Beige	Solid
P8	Pole	150	9 ± 2	Purple	13 ± 4	Yellow	Red	Semi curved	Small	Kidney	Yellow	Solid
P9	Pole	290	9 ± 1	White	19 ± 5	Yellow	Purple	Semi curved	Medium	Kidney	Other	Solid
P10	Pole	340	10 ± 2	White	19 ± 3	Green	Purple	Semi curved	Medium	Kidney	White	Solid
P12	Pole	233	11 ± 2	White	12 ± 2	Yellow	Red	Straight	Medium	Rounded	Beige	Hilum spot
P13	Bush	54	10 ± 2	White	11 ± 2	Yellow-green	Solid	Semi curved	Medium	Rounded	Beige	Hilum spot
P14	Pole	310	11 ± 2	Purple	13 ± 2	Yellow-green	Solid	Semi curved	Medium	Rounded	White	Hilum spot
P15	Pole	42	9 ± 2	Purple	12 ± 2	Yellow-green	Solid	Straight	Medium	Elongated	White	Hilum spot
P16	Pole	229	9 ± 3	White	12 ± 2	Yellow-green	Solid	Semi curved	Large	Rounded	White	Hilum spot
P19	Half-runner	197	9 ± 2	White	14 ± 3	Yellow-green	Solid	Straight	Medium	Elongated	White	Hilum spot
P24	Half-runner	160	9 ± 2	White	8 ± 1	Yellow-green	Purple	Straight	Medium	Rounded	White	Hilum spot
P26	Pole	286	9 ± 2	Purple	16 ± 3	Yellow-green	Purple	Curved	Medium	Kidney	Beige	Mottled
P28	Pole	265	10 ± 2	Purple	11 ± 1	Yellow	Red	Semi curved	Medium	Rounded	Beige	Mottled
P29	Pole	330	10 ± 1	White	15 ± 1	Yellow	Red	Curved	Medium	Rounded	Purple	Mottled
P32	Pole	200	10 ± 5	Purple	12 ± 2	Yellow	Red	Semi curved	Medium	Rounded	Yellow	Mottled
P33	Half-runner	209	11 ± 2	Purple	17 ± 2	Green	Purple	Semi curved	Medium	Elongated	White	Mottled
P34	Bush	50	8 ± 1	White	12 ± 2	Yellow-green	Solid	Straight	Medium	Elongated	Brown	Mottled
P40	Half- runner	185	9 ± 2	Purple	5 ± 6	Yellow	Red	Semi curved	Large	Elongated	Brown	Solid
P41	Bush	45	10 ± 3	Purple	10 ± 1	Yellow	Red	Straight	Medium	Elongated	Yellow	Solid
P42	Bush	62	9 ± 3	Purple	13 ± 3	Yellow	Red	Semi curved	Medium	Elongated	Other	Solid
P43	Bush	62	11 ± 2	Purple	12 ± 2	Yellow	Purple	Straight	Medium	Elongated	Beige	Solid
P44	Bush	65	8 ± 2	Purple	10 ± 2	Yellow	Solid	Straight	Small	Elongated	Yellow	Hilum spot
P45	Bush	75	8 ± 2	Purple	8 ± 2	Yellow	Red	Straight	Small	Elongated	Black	Solid
P47	Pole	116	5 ± 1	White	8 ± 2	Green	Solid	Curved	Medium	Rounded	Beige	Hilum spot
P62	Bush	43	9 ± 1	Purple	12 ± 2	Yellow	Purple	Semi curved	Small	Rounded	Black	Solid
P64	Pole	320	10 ± 2	Purple	18 ± 3	Yellow	Purple	Semi curved	Medium	Kidney	Beige	Mottled
P73	Half-runner	185	10 ± 2	Purple	10 ± 1	Yellow	Red	Straight	Medium	Elongated	Black	Solid

Complete trait list available in Annex 5

ͥSeed size: Seeds classified according to their volume as large (greater than 1 cm³), medium (between 0.4 and 1 cm³), and small (less than 0.4 cm³)

Although trait comparison between seasons was not statistically conducted, qualitative field observations indicated consistent phenological behavior across gardens.

## Results

### Agrobotanical traits

**Fig. 3:**
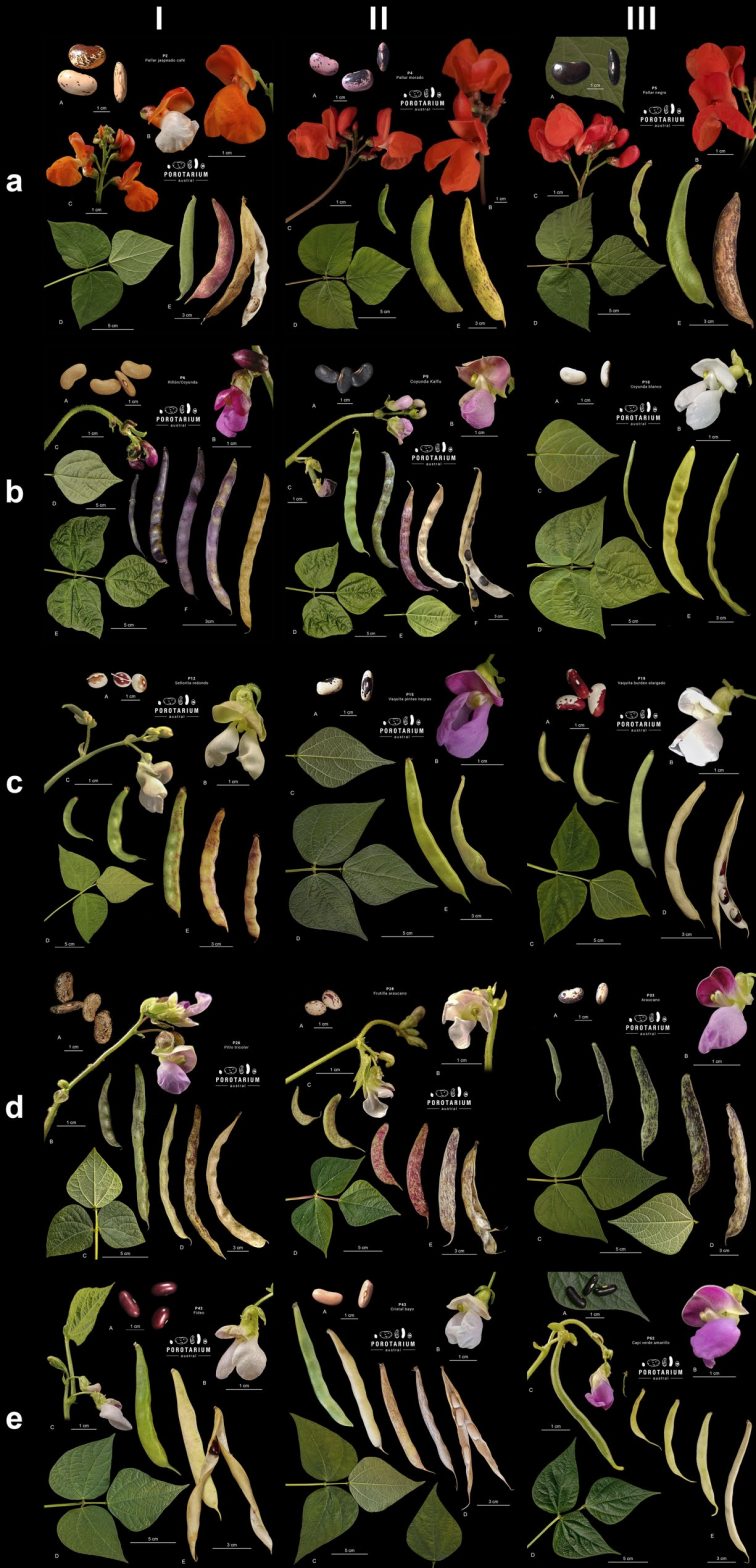
Lankester-style plates showing the morphology of six groupings of bean landraces. The Lankester plates were created from photographs taken during the 2022–2023 and 2023–2024 agricultural seasons, illustrating the morphology of the seed, flower, inflorescences, adaxial and abaxial surfaces of the leaf, and pod. The groupings generated from this work are: (**a**) Pallares, (**b**) Coyundas, (**c**) Hilum spot, (**d**) Mottled, (**e**) Solid. Individual images are available in *Annex* 6

We evaluated the traits of four morphological components: leaf, flower, pod, and seed for 30 bean landraces. There is a high degree of morphological variability in these traits, making each landrace unique, as can be observed in the plates for 15 of these landraces (Fig. 3). On the other hand, there are also similarities, which leads us to create subgroups among these landraces. The first trait we evaluated is the leaf blade. While all beans have trifoliate leaves, the differences lie in their size, blade shape (oval, lanceolate, cordate), apex (sharp or blunt), base (rounded, cordate, obtuse), texture (smooth, rough), pubescence (low, medium, high), venation (strongly, moderately, or lightly marked), volume (flat, cupped), and leaflet arrangement (perpendicular or angled).

All bean flowers share the same floral formula of two fused upper petals, two wings, one keel petal, and nine fused stamens with one free. Variability is observed in the color of the petals, which we categorized as: red, white, or purple, in the grouping of the inflorescence (simple or compound), and its position (axillary or apical).

The pod shows variation in the profile shape, for which we defined three categories: curved, moderately curved, and straight, as well as variation in the apex shape, for which we also defined three categories: curved, semi curved, and straight. Its main color also varies (green, yellowish-green, yellow), as well as the pattern (solid or mottled), color of the mottling if present (red or purple), seed arrangement in the pod (loose, embedded), and total length from base to tip. These traits are not fixed, as the pod changes throughout the plant’s phenological development (Fig. 3).

The seed holds much of the variability, and it is also where many of the names traditionally used to identify these landraces originate. We defined three main shapes: round, elongated, and kidney-shaped, each with three subcategories used for the botanical description of the seed. Also noteworthy are the color differences, which we categorized as: yellow, beige, white, brown, purple, black, and other (including blue and burgundy), along with its pattern (solid, hilum spot, or mottled) and the distribution of the pattern based on the seed structures (detailed in Appendix 1), whether centered around the hilum or evenly distributed across the seed surface. Additionally, we categorized the seeds by volume into large (greater than 1 cm³), medium (between 0.4 and 1 cm³), and small (less than 0.4 cm³). 

**Fig. 4:**
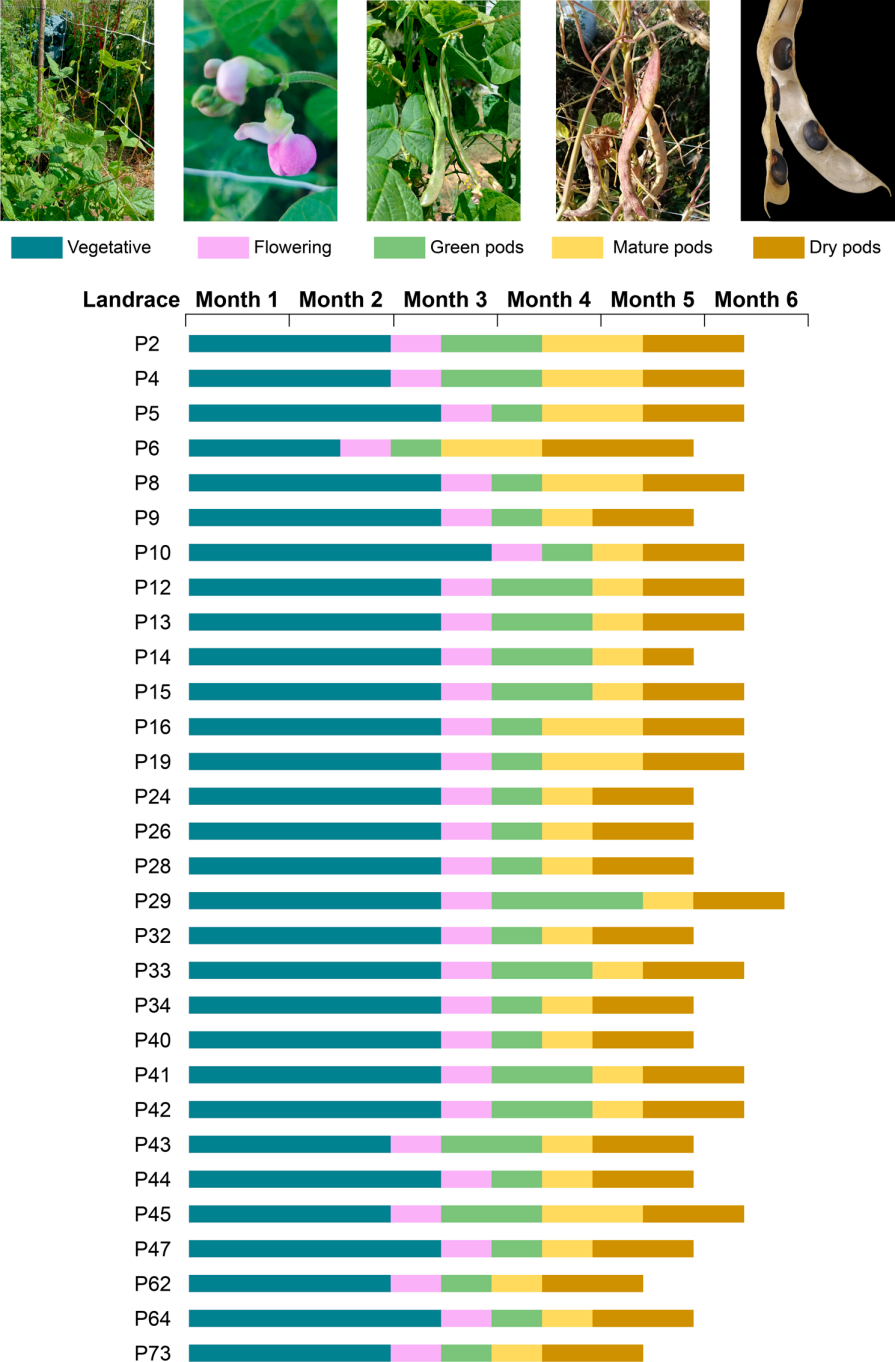
Phenology of traditional bean landraces. Vegetative growth, flowering, and pod development periods for green consumption, mature pods, and dry pods (for seed harvest and food saving) in 30 bean landrace landraces cultivated during the two PA agricultural seasons. Month 1 corresponds to November. Images show P009 PVG

We recorded the phenological development of each landrace between November and April (Fig. 4). On average, flowering occurs in January, and pod development from February to April, with deviations for each landrace. Exceptionally early landraces were observed (such as P6, Coyunda riñón) and others that were late (such as P29 Sortelino), which are traits of interest when selecting the landrace to cultivate in the garden.

### Trait relationships

Upon conducting the Principal Component Analysis on the data matrix (Table 1, Annex 5), the first two principal components explained 97.08% of the total variation (Annex 2). The principal component (PC) 1 was composed of plant height (0.369), internode distance (0.319), central leaflet width (0.376), and pod length (0.332), making it an indicator of overall plant size. PC2 was composed of seed length (-0.328), width (-0.349), depth (-0.296), volume (-0.383), and weight (-0.376). Therefore, it serves as an indicator of seed size and correlates with the small, medium, and large categories we created based on seed volume. Subsequently, the landraces were distributed in the plane formed by these two principal components, with PC1 on the X-axis and PC2 on the Y-axis. Landraces of the species *P. coccineus* (known as “Pallares”), corresponding to P2, P4, and P5, formed a group distinct from the *P. vulgaris* landraces. This distinction is primarily due to the large seed size of *P. coccineus* and the fact that all share a pole growth habit. Conversely, within the *P. vulgaris* landraces, smaller seeds result in smaller plants, whereas medium-sized seeds can produce plants of all sizes and growth habits, encompassing a wide variability. 

**Fig. 5:**
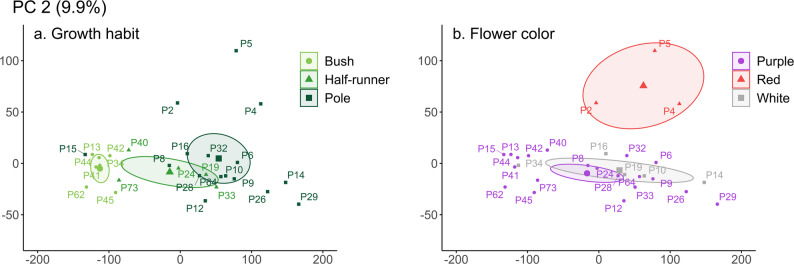
Grouping of traditional bean landraces based on (**a**) growth habit and (**b**) flower color. Thirty traditional bean landraces are spatially distributed according to their quantitative traits: growth habit (X-axis; principal component 1) and seed size (Y-axis; principal component 2). The colors and ellipses represent the groupings of these landraces with respect to (**a**) growth habit and (**b**) flower color

The growth habit (Fig. 5a) is directly related to plant size; pole plants are the tallest, half-runner plants are of intermediate height, and bush plants are the shortest. This information confirms that these concepts are closely tied to plant size. There is also a clear distinction between species, as the three largest seeds of *P. coccineus* all belong to pole plants, whereas the *P. vulgaris* landraces exhibit much less dispersion among themselves and are evenly distributed across bush, half-runner, and pole growth habits. The same pattern is observed in the analysis of flower color (Fig. 5b), where a clear clustering of red flowers is evident, associated with *P. coccineus* plants. It is worth noting that *P. coccineus* can also exhibit other flower colors, as seen in the “pallar blanco” landrace, which has white flowers matching its seed color. However, despite the presence of this landrace in the PA Seed Refuge, it was not included in the analysis due to its low sample size, as it encountered cultivation challenges in some gardens.

On the other hand, the *P. vulgaris* landraces exhibited white and purple flowers, which were evenly distributed among landraces of different plant and seed sizes, but did not display red flowers. However, a phenomenon of hybridization between the two species, *P. coccineus* and *P. vulgaris*, was observed, resulting in flowers pink to magenta in color. These flowers, in turn, produced pods and seeds with hybrid traits. This phenomenon occurred only in isolated plants and not across the entire crop, making it inconsistent and therefore it was not included in these analyses. During the phenological development of the pod, its color, pattern, curvature, and size vary significantly as it matures. This is when the most evident and striking morphological changes are observed. For instance, during the immature pod stage, most pods are green (commonly referred to as “*porotos verdes”*, green beans, for consumption), with some exceptions being yellow. Conversely, once dried, all pods acquire a light brown color. Therefore, we decided to analyze the pod traits at its intermediate stage, when it contains developing seeds, referred to as the mature or “granada” pod. 

**Fig. 6:**
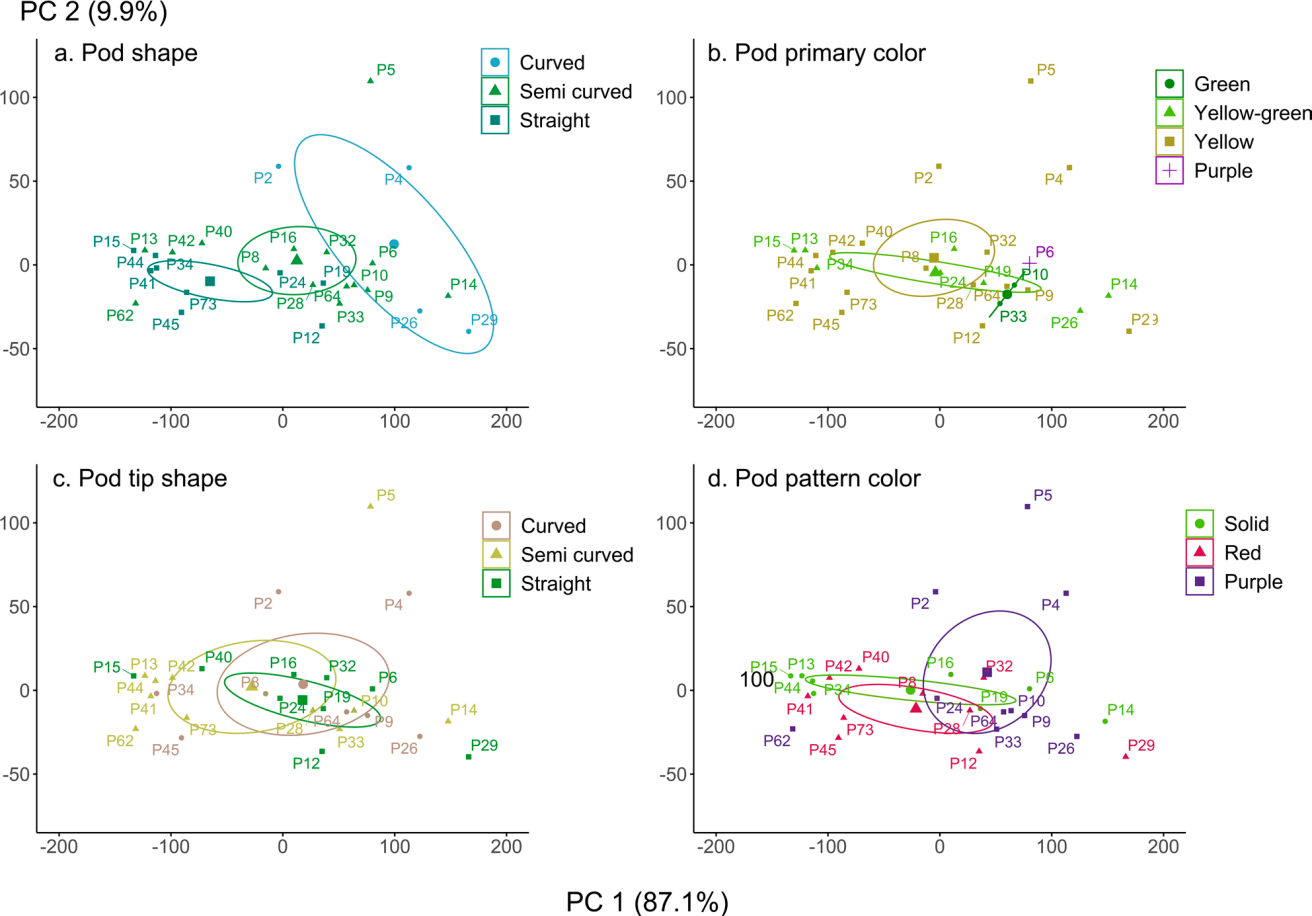
Grouping of traditional bean landraces based on pod traits. Thirty traditional bean landraces are presented, spatially distributed according to their quantitative traits: plant size (X-axis; principal component 1) and seed size (Y-axis; principal component 2). Colors and ellipses illustrate the grouping of these landraces based on their: (**a**) Pod shape, (**b**) Pod color, (**c**) Pod tip shape, and (**d**) Pod pattern color. Detailed descriptions of the pod and tip shape categories are provided in Annex 1

Regarding the shape of the pod (Fig. 6a), smaller plants exhibited straight pods, medium-sized plants had semi curved pods, and the larger plants presented curved pods. No groupings were observed in pod color (Fig. 6b), as green, yellow-green, and yellow pods were distributed evenly, with no relation to plant or seed size. However, the three landraces of *P. coccineus* have yellow pods in their mature state, while the fully purple pod was only observed in one landrace, P6 with a pole growth habit. Therefore, this is not a relevant criterion for grouping the landraces of *P. vulgaris*. There is a wide dispersion in the shape of the mature pod tip (Fig. 6c). It does not correlate with either of the two principal components. The color of the pod pattern is evenly distributed across different species (Fig. 6d). The pattern is purple in larger plants with larger seeds, including the three *P. coccineus* landraces, while medium or small plants with smaller seeds exhibit patterns in reddish tones or a uniform solid color with no pattern. 

**Fig. 7:**
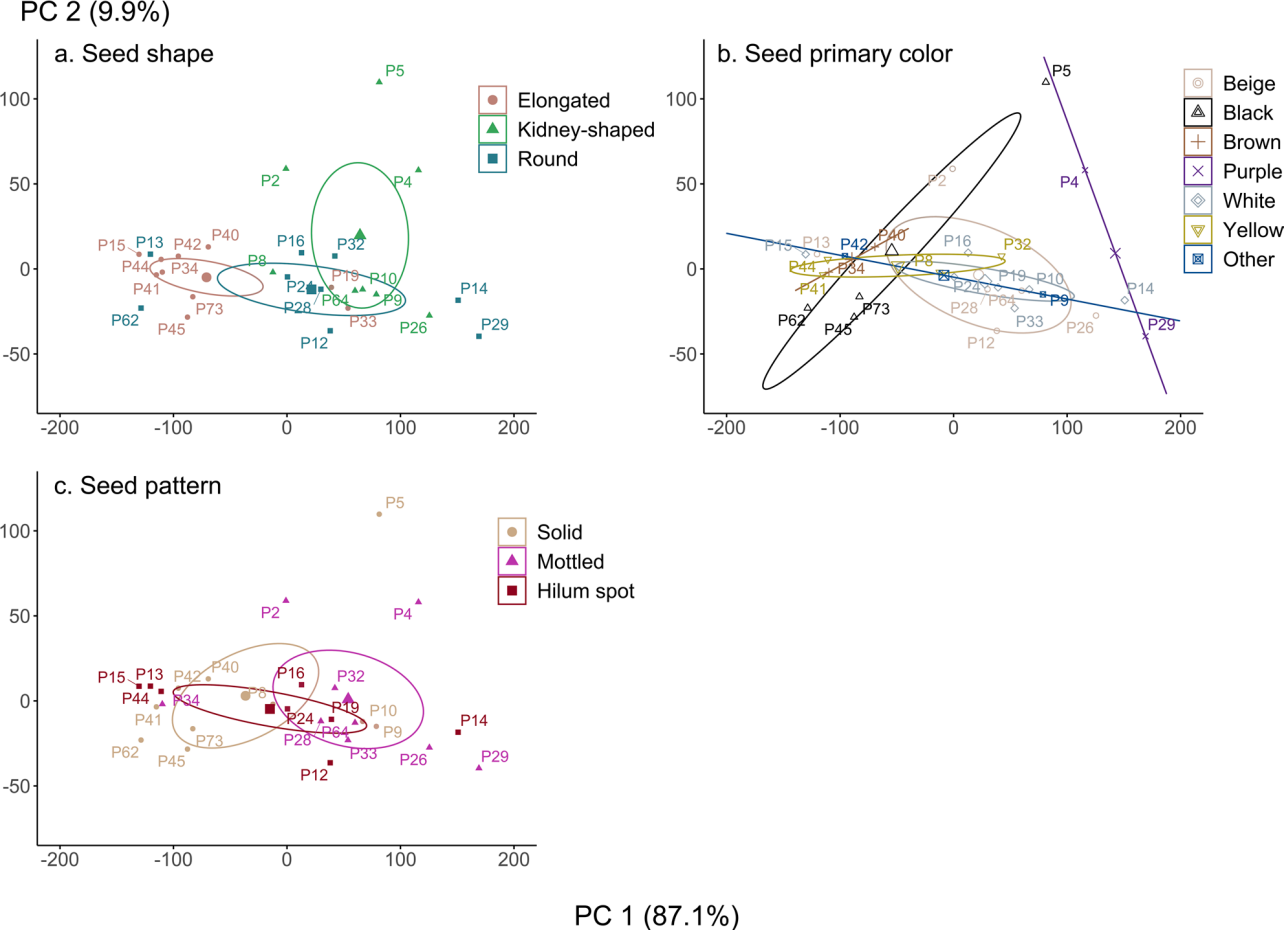
Grouping of traditional bean landraces based on seed traits. A total of 30 traditional bean landraces are spatially distributed according to their quantitative traits: plant size (X-axis; principal component 1) and seed size (Y-axis; principal component 2). Colors and ellipses indicate groupings of these landraces based on their (**a**) seed shape, (**b**) seed primary color, and (**c**) seed pattern. Detailed descriptions of seed shape categories are provided in Annex 1

Regarding seed shape (Fig. 7a), kidney-shaped seeds are generally large and belong to larger plants with pole growth habits, such as *Pallares* and *Coyundas* (P6, P9, P10). Among the remaining landraces of *P. vulgaris*, seeds with round and elongated shapes were small or medium-sized. Elongated seeds are associated with smaller plants with bush growth habits, while round seeds result in plants of various growth habits and sizes.

It is important to note that complex seed shapes were grouped into these three categories to facilitate their study; however, subcategories within these groups can also be identified (Annex 1b). Despite both being kidney-shaped, *P. coccineus* exhibits thick kidney-shaped seeds, while *Coyundas* have thin kidney-shaped seeds. Thin elongated seeds produce bush plants without a string, suitable for consumption as green beans, whereas thick elongated seeds, such as those of *Señorita*,* Freire*, and *Azufre*, are primarily consumed as dry seeds. Spherical round seeds include *Señorita redondo* and *Manteca*, which also have a characteristic pod shape with the round seeds clearly visible inside. In contrast, oval round seeds include *Caballero*, which are much larger. *P45 Cargamento* exhibits a unique, characteristic angular elongated shape. The seed color (Fig. 7b) is evenly distributed among plants and seeds of various sizes.

### Groupings

A marked interspecific difference was observed between *P. coccineus* and *P. vulgaris*. The species *P. coccineus* (*Pallares* group) is characterized by pole growth, broad leaves, red flowers, thick yellow, mottled mature pods, and large kidney-shaped seeds with an average 100-seed weight of 145 g. Among the 27 landraces of *P. vulgaris* in this study, we propose four categories based on their diversity of agrobotanical traits: Coyundas, Hilum-Spotted, Mottled, and Solid (Fig. 3).

*Coyundas* are characterized by a pole growth habit, heart-shaped leaves with convex curvature on the adaxial surface, long, wide, curved pods (from which their common name derives, resembling the long leather strap used to tie oxen yokes), and thin kidney-shaped seeds with a uniform solid color, averaging 100-seed weight of 60 g. The *Hilum-Spotted* group has elongated leaves, solid pods with no patterns, and seeds with large spots around the hilum, are medium sized, with an average weight of 100-seed weight of 54 g. This group includes beans commonly referred to as *“Señoritas”* (ladies) due to their resemblance to the vulva or *“Vaquitas”* (little cows) for their spots. They have varied growth habits and pod shapes, which can range from straight to moderately curved. The *Mottled* group is characterized by half-runner or pole growth habits, mottled patterns covering both their mature pods and seeds, which are medium sized, with an average 100-seed weight of 54 g. They also have variable leaf and pod shapes. The *Solid* group displays a bush growth habit, teardrop-shaped leaves, thin pods with slight mottling, and solid colored beans. These seeds are small to medium-sized, with an average 100-seed weight of 42 g. Variation in pod shape is observed within this group. We observed greater phenological variability within the *Pallares* and *Coyundas* groups, whereas the *Hilum-Spotted*, *Mottled*, and *Solid* groups (all belonging to *P. vulgaris*) are more uniform, with flowering occurring in late January and dry seed harvest in late March.

## Discussion

This study presents an important contribution to the documentation of bean landraces in southern South America, Chile. We show that the observed agrobotanical and phenological diversity reflects the rich evolutionary history and cultural relevance of these landraces in the Southern Andes. Beyond morphological characterization, the in vivo approach of this study allowed us to explore both ecological and social dimensions of bean conservation under real cultivation conditions. The gardeners who participated in the Porotarium Austral Seed Refuge shared observations on differences in plant performance, frost resistance, and productivity across sites, showing how local microclimates and traditional management influence the expression of traits. Many of them expressed their willingness to continue cultivating preferred landraces for household consumption and future seed exchanges (*trafkintu*), demonstrating that in vivo conservation is not only possible but naturally embedded in their everyday practices.

The distinct morphological patterns underscore the diversity found in these landraces, with their individual traits being a key element of their cultural significance in traditional Andean agriculture, and suggesting potential adaptations to different pressures, such as leaf venation and pubescence, which supporting findings from Fang and Xiong (2015) on drought and frost resistance mechanisms in legumes. Flower morphology was consistent across landraces, but the variation in petal color and inflorescence structure may be linked to pollination strategies and genetic diversity. The clustering of red flowers in *P. coccineus* aligns with previous reports (López-Báez et al. 2018; Mendoza et al. 2006), while the presence of white flowers in “pallar blanco” highlights intraspecific variation within this species. Other studies have also reported white and purple flowers for this species (Arriagada et al. 2021).

The strong correlation between pod traits and phenological stages suggests that selection for certain pod characteristics may enhance green pod consumption or seed yield. The observed hybridization between *P. coccineus* and *P. vulgaris*, though inconsistent, supports prior findings (Bitocchi et al. 2017; Singh 2001) on interspecific crosses. However, the limited fertility of these hybrids suggests genetic barriers to introgression, maintaining species integrity. The shape of the pod aligns with the observations of Mendoza et al. (2006), who describe that both *P. coccineus* and *P. vulgaris* primarily have semi-curved pods, followed by curved ones. No relationship with seed size was observed. Therefore, this trait is more closely related to plant size than to differences between species.

Significant variability in bean seed color was observed, consistent with findings from other studies on this genus (López-Báez et al. 2018; Mendoza et al. 2006). Regarding seed pattern (Fig. 7c), a slight trend was noted: mottled seeds tend to be associated with larger plants, while solid and hilum spot seeds are evenly distributed among medium- and small-sized plants. This trait is unrelated to seed size. Most mottled and solid seeds (90%) originate from purple- or red-mottled pods, whereas seeds with hilum spots primarily (78%) originate from solid-colored pods. Seed morphology, particularly shape and volume, was a key determinant of landrace classification. The PCA results confirm that plant size and growth habit are intrinsically linked, with *P. coccineus* consistently producing the tallest plants and largest seeds. These findings reinforce the role of genetic factors in growth habit determination while highlighting the phenotypic plasticity of *P. vulgaris* landraces. It is important to consider that part of the variability observed in this study may be related to external factors, including the geographic location, layout, orientation, and soil type of the garden, as well as the agricultural management techniques employed by each gardener. During the study, we also observed changes in the reported growth habit and common names compared to initial cadastres, underscoring the importance of documenting and cataloging these landraces, while considering the phenotypic plasticity of the species.

A culminating milestone in this research was the *Huerta-Museo Porotarium Austra*l, held in February 2024 in the municipality of Pucón. Conceived as a transdisciplinary living exhibition, it brought together forty cultivated landraces, the community seed library (*Semilloteca Porotarium Austral*), and a public program intertwining science, art, and agriculture. The gardeners themselves acted as guides of the museological route, welcoming visitors from across Chile — including farmers, local authorities, and tourists — and sharing cultivation experiences, stories, and recipes associated with each landrace. This collective and sensory space became a meeting point for exchange, learning, and appreciation of agrobiodiversity. During the study period, more than three hundred farmers from Santiago to Chiloé participated in seed exchanges through *La Colección Portera* — a set of five randomly selected landraces distributed for cultivation — thus expanding the living network of in vivo conservation far beyond the initial study area. The Porotarium Austral experience demonstrates the value of combining scientific documentation with community stewardship. By linking women’s homegardens, seed-exchange practices, and morphological analysis, this work strengthens regional seed sovereignty and contributes to agrobiodiversity policies that recognize in vivo conservation as a viable and inclusive strategy for climate adaptation, food security, and biocultural heritage conservation.

## Conclusions

We studied 30 out of the 90 landraces present in the Porotarium Austral Seed Refuge as of 2023, so it would be important to gather more information in the future from a larger number of landraces, also including other species such as *P. lunatus*. However, we observed that for our study area in the Andean La Araucanía region of southern Chile, *P. vulgaris* is the species of greatest importance in terms of its diversity, and it is distinguished from *P. coccineus* through multiple morphological traits of its seed, plant, flower, and pod. These results highlight that the family farm gardens in the Andean La Araucanía (Wallmapu) serve as reservoirs for a great diversity of bean landraces, constituting biocultural refuges of high biocultural value.

We observed several other traits of interest, such as differences in sensitivity and resistance to frost and pests at different stages (Annexes 3 and 4), which are also expressed through diverse recipes and cooking methods, emphasizing the cultural and social importance of beans as traditional food. This local diversity is crucial for addressing challenges such as climate change, helping to combat poverty and malnutrition, and improving quality of life (Mirsha et al., 2024). Understanding the traits of these landraces also allows us to cultivate, share, and consume them while conserving their diversity, health benefits, while strengthening both social-ecological resilience and the commons in the southern Andes (Ibarra et al. 2023, 2024a).

In summary, this study documents, for the first time, the morphological and phenological traits of thirty bean landraces cultivated in southern Chile through a participatory in vivo approach. The remarkable variability observed across landraces highlights the role of women-led homegardens as biocultural refuges and living laboratories for agrobiodiversity. Strengthening these networks can support regional strategies for seed conservation, promote dietary diversity, and foster adaptation to climate change. By cultivating, sharing, and valuing these landraces, communities in the southern Andes reaffirm their autonomy and capacity to safeguard agricultural biodiversity for future generations.

## Supplementary information

Below is the link to the electronic supplementary material.

Supplementary Material 1

## Data Availability

The dataset generated during the current study is partially available in the social media (Instagram and Spotify) of Porotarium Austral @porotorium_austral. And full data are available from the corresponding author on reasonable request.

## References

[CR1] Afshin A, Sur PJ, Fay KA, Cornaby L, Ferrara G, Salama JS, Murray CJ (2019) Health effects of dietary risks in 195 countries, 1990–2017: a systematic analysis for the global burden of disease study 2017. Lancet 393(10184):1958–1972 30954305 10.1016/S0140-6736(19)30041-8 PMC6899507

[CR3] Akinola R, Pereira LM, Mabhaudhi T, De Bruin FM, Rusch L (2020) A review of Indigenous food crops in Africa and the implications for more sustainable and healthy food systems. Sustainability 12(8):3493 33520291 10.3390/su12083493 PMC7116648

[CR2] Arriagada O, Schwember AR, Greve MJ, Urban MO, Cabeza RA, Carrasco B (2021) Morphological and molecular characterization of selected Chilean runner bean (*Phaseolus coccineus* L.) genotypes shows moderate agronomic and genetic variability. Plants 10(8):1688 34451733 10.3390/plants10081688 PMC8400864

[CR4] Baginsky C, Ramos L (2018) Situación de Las legumbres En chile: Una Mirada agronómica. Revista Chil De nutrición 45:21–31

[CR5] Barreau A, Ibarra JT, Wyndham F, Kozak R (2019) Shifts in Mapuche food systems in southern Andean forest landscapes: historical processes and current trends of biocultural homogenization. Mt Res Dev 39(1)

[CR6] Bascur GB, Tay JU (2005) Collection, characterization and use of genetic variation in Chilean bean germplasm (*Phaseolus vulgaris* L.) 1. Agricultura Técnica 65(2):135–146

[CR7] Beany things (2024) about us. Retrieved 27 Jan 2025, from: https://beanythings.wordpress.com/about/

[CR8] Biodiversidad A & CONADI (2020) Catastro, reconocimiento y descripción de las semillas tradicionales mapuche de la región de la araucanía. región de la araucanía, Chile. Retrieved 27 Jan 2025, from: https://www.biodiversidadalimentaria.cl/wp-content/uploads/2020/07/Catastro_semillas_tradicionales_La_Araucan%C3%ADa.pdf

[CR10] Bitocchi E, Rau D, Bellucci E, Rodriguez M, Murgia ML, Gioia T, Papa R (2017) Beans (Phaseolus ssp.) as a model for Understanding crop evolution. Front Plant Sci 8: 722 28533789 10.3389/fpls.2017.00722 PMC5420584

[CR9] Bocci R, Chable V (2009) Peasant seeds in europe: stakes and prospects. J Agric Environ Int Dev (JAEID) 103(1/2):81–93

[CR12] Bria EJ, Suharyanto E, Purnomo P (2019) Variability and intra-specific classification of Lima bean (Phaseolus lunatus L.) from Timor Island based on morphological characters. J Trop Biodivers Biotechnol 4(2):62–71

[CR11] Börner A, Khlestkina EK (2019) Ex-situ genebanks—seed treasure chambers for the future. Russian J Genet 55: 1299–1305

[CR13] Broughton WJ, Hernández G, Blair M, Beebe S, Gepts P, Vanderleyden Jos (2003) Beans (Phaseolus spp.) - Model food legumes. Plant Soil 252: 55–128

[CR14] Campbell R, Roa C, Santana-Sagredo F (2018) Más sureño que los porotos: primeros fechados ^14^C AMS para el sitio Cueva de los Catalanes. Boletín de la Sociedad Chilena de Arqueología vol 48 pp. 85–89

[CR15] Cardona C (2004) Common beans-latin america. Crop post-harvest: Sci Technol, 145–150

[CR16] Ceccon E (2008) La revolución verde: tragedia En Dos actos. Ciencias 91: 21–29

[CR17] Christina GR, Thirumurugan T, Jeyaprakash P, Rajanbabu V (2021) Principal component analysis of yield and yield related traits in rice (Oryza sativa L.) landraces. Electron J Plant Breed 12: 907–911

[CR18] Debouck DG, Hidalgo R (1985) Morfología de la planta de frijol común. En: López Genes, Marceliano; Fernández O., Fernando O.; Schoonhoven, Aart van (eds.). Frijol: investigación y producción. Programa de las Naciones Unidas (PNUD); Centro Internacional de Agricultura Tropical (CIAT), Cali, CO. pp. 7–41

[CR19] Didinger C, Gutekunst K, Greenhalgh-Ball A, Mbelenga E, Newnham P, Bean Science and Innovation Advisory Council (2024) &. Why are beans so brilliant? Accedido el 9 de octubre de 2024. Retrieved 27 Jan 2025. https://sdg2advocacyhub.org/beans-is-how/resources/

[CR20] Diulgheroff S, Robinson J (2009) El estado de la conservación ex situ. En: Segundo Informe sobre el Estado de los Recursos Fitogenéticos para la Alimentación y la Agricultura en el mundo. Roma, Italia. Retrieved 27 Jan 2025. https://www.fao.org/4/i1500s/i1500s00.htm

[CR21] Dulloo E, Jarvis D, Thormann I, Scheldeman X, Salcedo J, Hunter D, Hodgkin T (2009) El estado de la ordenación in situ. En: El Segundo informe sobre el estado de los recursos fitogenéticos para la alimentación y la agricultura en el mundo. Roma, Italia. Retrieved 27 Jan 2025. https://www.fao.org/4/i1500s/i1500s00.htm

[CR22] Espeleta ALG, Moraga FM (2011) El Grito de Los Bienes comunes: ¿qué son? Y ¿qué Nos aportan? Revista de Ciencias Sociales, pp. 131–132

[CR23] Fang Y, Xiong L (2015) General mechanisms of drought response and their application in drought resistance improvement in plants. Cell Mol Life Sci 72: 673–689 25336153 10.1007/s00018-014-1767-0 PMC11113132

[CR24] Food and Agriculture Organization. FAO/INFOODS (2024) Global food composition database for pulses version 1.0 (uPulses1.0). Retrieved Oct 8 2024. from: http://www.fao.org/3/a-i6832e.pdf

[CR25] Gazan R, Maillot M, Reboul E, Darmon N (2021) Pulses twice a week in replacement of meat modestly increases diet sustainability. Nutrients 13(9): 3059 34578936 10.3390/nu13093059 PMC8466503

[CR26] Global Bean Project (2025). About the Global Bean Project https://www.globalbean.eu/about/

[CR27] Gómez-Campo C (2006) Erosion of genetic resources within seed genebanks: the role of seed containers. Seed Sci Res 16(4): 291–294

[CR28] Grajales GIC (2010) El Conocimiento tradicional y El concepto de territorio. Núcleo De Estudios Pesquisas E Projectos De Reforma Agrária, 1–12

[CR29] Granado FS, Maia EG, Mendes LL, Claro RM (2021) Reduction of traditional food consumption in Brazilian diet: trends and forecasting of bean consumption (2007–2030). Public Health Nutr 24(6): 1185–1192 33314999 10.1017/S1368980020005066 PMC10195620

[CR30] Havemeier S, Erickson J, Slavin J (2017) Dietary guidance for pulses: the challenge and opportunity to be part of both the vegetable and protein food groups. Ann N Y Acad Sci 1392(1): 58–66 28146277 10.1111/nyas.13308

[CR31] Ibarra JT, Barreau A (2019) Huertas familiares Como refugios bioculturales: espacios femeninos Para cuestionar Los desbalances de poder y construir soberanía alimentaria. Diálogos 8(14): 46–47

[CR34] Ibarra JT, Caviedes J, Barreau A (2024b) Homegardens in the Southern andes: cultivating agrobiodiversity, learning, and sovereignty from interculturality. Ch. 5. In: Gagnon T, Nazarea V (eds) Embodying biodiversity: sensory conservation as refuge and sovereignty. University of Arizona, Tucson, USA, pp. 131–151

[CR32] Ibarra JT, Caviedes J, Barreau A, Pessa N (2019) Huertas familiares y comunitarias: cultivando soberanía alimentaria. Ediciones universidad católica de Chile, Santiago, Chile. 228 pp. ISBN: 978-956-14-2331-2

[CR33] Ibarra JT, Caviedes J, Monterrubio-Solís C, Barreau A, Marchant C (2024a) Social-ecological resilience: knowledge of agrobiodiversity by Campesinos and migrants in the face of global changes. J Environ Manage 370: 122461 39265494 10.1016/j.jenvman.2024.122461

[CR35] Ibarra MI, Guasch A, Ojeda J, Riquelme W, Ibarra JT (2023) Commons of the south: ecologies of interdependence in local territories of Chile. Sustainability 15: 10515

[CR36] Ickowitz A, Powell B, Rowland D, Jones A, Sunderland T (2019) Agricultural intensification, dietary diversity, and markets in the global food security narrative. Global Food Secur 20: 9–16

[CR37] Iglesias M, Maciel M, Orozco R (1992) Caracterización agronómica de 42 genotipos de frijol (*Phaseolus* vulgaris) en el municipio de Zapopan, jal. Tesis Profesional, Facultad de Agronomía, Universidad de Guadalajara

[CR38] Jackson LE, Brussaard L, de Ruiter PC, Pascual U, Perrings C, Bawa K (2013) Agrobiodiversity Encyclopedia Biodivers 126:135

[CR39] JUNAEB (2024) Menú basado en plantas: La Iniciativa de JUNAEB para incentivar el consumo de granos, legumbres, frutas y verduras. Retrieved 15 Oct 2024 from: https://www.junaeb.cl/menu-basado-en-plantas-la-iniciativa-de-junaeb-para-incentivar-el-consumo-de-granos-legumbres-frutas-y-verduras/

[CR40] Kastler G, Onorati A, Brac B (2013) Seeds and peasant autonomy. Right Food Nutr Watch, 47–53

[CR41] Khoury CK, Bjorkman AD, Dempewolf H, Ramirez-Villegas J, Guarino L, Jarvis A, Struik PC (2014) Increasing homogeneity in global food supplies and the implications for food security. Proc natl Acad Sci, 111(11): 4001–4006 10.1073/pnas.1313490111 PMC3964121 24591623

[CR42] Kushi LH, Meyer KA, Jacobs DR Jr (1999) Cereals, legumes, and chronic disease risk reduction: evidence from epidemiologic studies. Am J Clin Nutr 70(3): 451S–458S 10479217 10.1093/ajcn/70.3.451s

[CR44] Li DZ, Pritchard HW (2009) The science and economics of ex situ plant conservation. Trends Plant Sci 14(11): 614–621 19818672 10.1016/j.tplants.2009.09.005

[CR45] López-Báez LI, Taboada-Gaytán OR, Gil-Muñoz A, López PA, Ortiz-Torres E, Vargas-Vázquez MLP (2018) & Díaz-Cervantes R Diversidad morfoagronómica del frijol ayocote en el altiplano centro-oriente de Puebla. Revista Fitotecnia Mexicana, 41, 487–497

[CR43] Lê S, Josse J, Husson F (2008) FactoMineR: an R package for multivariate analysis. J Stat Softw 25: 1–18

[CR46] Manly BFJ, Navarro JA (2016) Multivariate statistical methods: a primer. Chapman and Hall, New York. 10.1201/9781315382135

[CR47] Mendoza MC, Vallejo PR, González FC, Colín SM (2006) Diversidad morfológica de poblaciones Nativas de Frijol común y Frijol Ayocote Del Oriente Del Estado de México. Revista Fitotecnia Mexicana 29: 111–119

[CR48] Mies M, Shiva V (1998) La praxis Del ecofeminismo: biotecnología, consumo y reporoducción. Icaria Editorial, vol 128

[CR49] Milano S, Ponzio R, Sard P (2018) El Arca del Gusto. Cómo construir el más grande catálogo de los sabores del mundo: un patrimonio a descubrir y salvar. Fundación Slow Food para la Biodiversidad https://www.fondazioneslowfood.com/wp-content/uploads/2021/05/SPA_libretto_arca-BASSA.pdf

[CR50] Ministerio de Agricultura (2024) Resolución 162 de 2024 Reconoce la existencia de semillas tradicionales y establece orientaciones al efecto. https://www.diariooficial.interior.gob.cl/publicaciones/2024/04/09/43821/01/2476151.pdf

[CR51] Mishra, R., Amrate, P. K. (2024). Genetic diversity in crop improvement: a cornerstone for sustainable agriculture and global food security. In Advances in plant biotechnology (Vol. 1, pp. 1–21). Cornous Publications LLP.

[CR52] Mohebodini M, Sabaghnia N, Behtash F, Janmohammadi M (2017) Principal component analysis of some quantitative and qualitative traits in Iranian spinach landraces. In Proceedings of the latvian academy of sciences. Sect B: Natl, Appl Sci. (Vol. 71, No. 4, pp. 307–310)

[CR53] Nazarea VD (2006) Cultural memory and biodiversity. Tucson, University of Arizona

[CR54] Nyau V (2014) Nutraceutical perspectives and utilization of common beans (Phaseolus vulgaris L.): A review. Afr J Food Agric Nutr Dev 14(7): 9483–9496

[CR55] Oduoye MO, Yusuf HA, Faloye TO, Ubechu SC, Chukwudile BU, Abdullahi AN, Paras P (2024) Challenges associated with the nutritional status of traditional and Indigenous foods in the global South. Food safety and quality in the global South Springer Nature Singapore. pp. 661–683

[CR57] Orlich MJ, Jaceldo-Siegl K, Sabaté J, Fan J, Singh PN, Fraser GE (2014) Patterns of food consumption among vegetarians and non-vegetarians. Br J Nutr 112(10): 1644–1653 25247790 10.1017/S000711451400261X PMC4232985

[CR58] Pan Africa Bean Research Alliance (2022) Improving food security, nutrition, incomes, natural resource base and gender equity for better livelihoods of smallholder households. Results to date (2015–2020). [Infographic] https://cgspace.cgiar.org/items/306cd212-12ce-40f8-b6ff-727b2684ddf1

[CR59] Pastor S, Gil A (2014) Procesos de domesticación y dispersión de La agricultura En El Sur de Sudamérica. Revista Española De Antropología Americana 44(2):453–464

[CR60] Peralta C, Thomet M, Celis A (2013) Curadoras de semillas. El Arte de conservar Las semillas de Los pueblos. Ediciones CETSUR

[CR61] Perelmuter T (2014) Bienes comunes vs. mercancías: Las semillas En disputa. Un análisis sobre Del Rol de La propiedad intelectual En Los actuales procesos de cercamientos. Sociedades Rurales producción Y Medio Ambiente 22: 53–86

[CR62] Peres S (2016) Saving the gene pool for the future: seed banks as archives. Stud History Philos Sci Part C: Stud History Philos Biol Biomedical Sci 55: 96–104 10.1016/j.shpsc.2015.09.002 26411896

[CR63] Pinheiro A, Ivanovic C, Rodríguez L (2018) Consumo de legumbres En Chile. Perspectivas y desafíos. Revista Chil De nutrición 45: 14–20

[CR64] Polanyi, K. (2001). The great transformation: The political and economic origins of our time (2nd ed.). Beacon Press.

[CR65] Qualset C, McGuire P, Warburton M (1995) Agrobiodiversity: key to agricultural productivity. Calif Agric 49(6): 45–49

[CR66] Qualset CO, Damania AB, Zanatta ACA, Brush SB (1997) Locally based crop plant conservation. Plant genetic conservation: the in situ approach. Springer Netherlands, Dordrecht, pp. 160–175

[CR67] R Core Team (2023) A language and environment for statistical computing. R foundation for statistical computing, Vienna, Austria. https://www.R-project.org/

[CR56] Órdenes-Abarca E, Sepúlveda-Cuevas T, Mellado-Ñancupil C (2023) Semillas tradicionales del Pueblo Mapuche*.* Santiago de Chile, FAO y MINAGRI. 10.4060/cc7173es

[CR68] Red de Guardianes de Semillas (2024) Diferencias entre semillas. https://redsemillas.org/publicaciones/

[CR69] Roa C, Silva C, Campbell R (2015) El aporte de la Isla Mocha al conocimiento sobre el aprovechamiento de plantas con valor alimenticio en el Sur de Chile (1000–1700 dC). En *Actas del XIX Congreso Nacional de Arqueología Chilena* (pp. 549–559). Universidad de Tarapacá Arica

[CR70] Rodríguez CH (2023) Soberanía de semillas campesinas y justicia climática en un mundo biotecnológico. Debates en Sociología, (57): 137–161

[CR71] Rogers DL (2004) Genetic erosion: no longer just an agricultural issue. Native Plants J 5(2): 112–122

[CR86] Salazar G, Reyes M, Kaulen-Luks S, Barrera MG, Burgos A, Ibarra JT (2025) Biocultural memory of reciprocity: the Mapuche trafkintu as social-ecological relationships of care and vindication. J Ethnobiology Ethnomedicine 21:59 10.1186/s13002-025-00811-2 PMC12382182 40866923

[CR72] Santillán TA (2018) Semillas campesinas, más que semillas botánicas. Ecofronteras, (64)5

[CR73] Semilla Austral (2023) Las semillas que cuidamos: Enciclopedia Etnobotánica por la Cooperativa Semilla Austral. Chile. Cuarta Edición. https://www.semilla-austral.coop/forms/las-semillas-que-cuidamos

[CR74] Singh SP (2001) Broadening the genetic base of common bean cultivars: a review. Crop Sci 41(6): 1659–1675

[CR75] Thompson HJ (2019) Dietary bean consumption and human health. Nutrients 11(12): 3074 31861044 10.3390/nu11123074 PMC6949954

[CR76] Thormann I, Engels JM (2015) Genetic diversity and erosion—A global perspective. Genetic Divers Eros Plants: Indic Prev, 263–294

[CR77] Trucchi E, Benazzo A, Lari M, Iob A, Vai S, Nanni L, Bertorelle G (2021) Ancient genomes reveal early Andean farmers selected common beans while preserving diversity. Nat Plants 7:123–128 33558754 10.1038/s41477-021-00848-7

[CR78] Tscharntke T, Clough Y, Wanger TC, Jackson L, Motzke I, Perfecto I, Whitbread A (2012) Global food security, biodiversity conservation and the future of agricultural intensification. Biol Conserv 151: 53–59

[CR79] UNDROP (2018) United Nations Declaration on the Rights of Peasants and Other People Working in Rural Areas. [Resolution 73/165]. https://undocs.org/en/A/RES/73/165

[CR82] Van de Wouw M, Kik C, van Hintum T, van Treuren R, Visser B (2010) Genetic erosion in crops: concept, research results and challenges. Plant Genetic Resour 8(1): 1–15

[CR81] Vargas-Vázquez P, Muruaga-Martínez JS, Martínez-Villarreal SE, Ruiz-Salazar R, Hernández-Delgado S & Mayek-Pérez N (2011) Diversidad morfológica del frijol ayocote del Carso Huasteco de México. Revista mexicana de biodiversidad. 82: 767–775

[CR80] Vargas-Vázquez P, Muruaga-Martínez JS, Pérez-Herrera P, Gill-Langarica HR, Esquivel-Esquivel G, Martínez-Damián MA & Mayek-Pérez N (2008) Caracterización morfoagronómica de la colección núcleo de la forma cultivada de frijol común del INIFAP. Agrociencia. 42: 787–797

[CR83] Vernooy R, Shrestha P, Sthapit B, Ramírez M (2016) Bancos comunitarios de semillas: orígenes, evolución y perspectivas. Bioversity International, Lima, Perú. Primera edición

[CR84] Visser B, Engels JM, Rao VR, Dempewolf J, Van D, Wouw M (2009) El estado de la diversidad. En: El segundo informe sobre el Estado de los recursos fitogenéticos para la alimentación y la agricultura en el mundo. Roma, Italia. Retrieved 27 Jan 2025, from: https://www.fao.org/4/i1500s/i1500s00.htm

[CR85] Watson C, Murphy-Bokern D (2022) Legumes translated report. Retrieved 27 Jan 2025, from: https://www.legumehub.eu/wp-content/uploads/2022/04/Legumes-Translated-Report-1-2.pdf

