# Supplementary material for: Colourful agrobiodiversity: morphology and phenology of bean landraces to face commodification of the commons in the southern Andes
Source: Bot Stud. 2026 Jan 15;67:1. doi: 10.1186/s40529-025-00488-6 (PMC12808010; doi:10.1186/s40529-025-00488-6)
Supplement: Supplementary file 1 — Supplementary Material 1 [file 40529_2025_488_MOESM1_ESM.zip › 40529_2025_488_MOESM1_ESM/40529_2025_488_MOESM22_ESM.docx]

**Annex 1. Morphological classification categories for bean pods and seeds.**

a. Pod shape and pod ship tape. Categories adapted from Iglesias et al. 1992, with some modifications. b. Bean seed shape, viewed with the hilum on top. Round, elongated, and kidney-shaped are the three main categories used in the analyses, while the nine subcategories were used for botanical descriptions. c. Bean seed structures in ventral view. The raphe is a ridge resulting from the fusion of the funiculus with the body of the ovule. The hilum is the scar formed by the connection with the pod. The micropyle is an opening through which the seed absorbs water. The hilum border and the area around the hilium are areas of interest for landrace seed description, as they hold a significant portion of the seed color variability.

**Annex 2. Contribution of quantitative bean variables to principal components.**

After conducting a principal component analysis, the composition of principal components 1 (PC1) and 2 (PC2) is shown based on the contribution of the quantitative variables measured in 30 bean landraces. The components with a contribution greater than 0.3 are highlighted in bold, indicating a significant contribution to the principal component.

**Annex 3. Landraces with traits of agrobotanical interest.**

**Annex 4. Records of the impact of animals and environmental conditions on beans during their development.**

Images taken as field records by farmers and researcher. a. Specimen of P044 PVM with leaf damage due to frost at the end of November, one month after germination, resulting in the loss of half of the crop. b. Leaf of P006 PVG after being bitten by *Phytotoma rara* on its right leaflet during vegetative growth. c. Vines of P006 PVG that fell to the ground after strong winds (*puelche*) during reproductive growth, resulting in losses in pod production.

**Annex 5. Detail of bean landraces traits.**

**Annex 6. Lankester plate P002 PCG - Pallar jaspeado café.** A. Seed; lateral and ventral views. The predominance of the mottled pattern is variable and can cover a large portion of the seed. B. Flower; in the two observed colorations: bicolor red with white and solid red. C. Inflorescence of solid red flower. D. Leaf; showing the upper surface of the lateral leaflets and the underside of the central leaflet. E. Pod; showing its three stages: immature (green), mature (“granada”), and dry (seed harvest). Each element is accompanied by its corresponding size scale. Photographic records obtained during agricultural seasons 1 and 2 of Porotarium Austral.

**Annex 7. Lankester plateP004 PCG - Pallar morado.** A. Seed; lateral and ventral views. The predominance of the mottled pattern is variable and can cover a large portion of the seed. B. Flower. C. Inflorescence. D. Upper surface of the leaf. E. Pod; showing three stages: young immature, immature (green), and mature(granada). Each element is accompanied by its corresponding size scale. Photographic records obtained during agricultural seasons 1 and 2 of Porotarium Austral.

**Annex 8. Lankester plate P005 PCG – Pallar negro.** A. Seed; lateral and ventral views. B. Flower. C. Inflorescence. D. Upper surface of the leaf. E. Pod; showing three stages: young immature, immature (green), and dry (seed harvest). Each element is accompanied by its corresponding size scale. Photographic records obtained during agricultural seasons 1 and 2 of Porotarium Austral.

**Annex 9. Lankester plate P006 PVG - Poroto riñón/Coyunda**. A. Seed; lateral and ventral views. B. Flower and bud. C. Inflorescence showing a pair of dark violet buds. D. Leaf; underside of the central leaflet. E. Whole leaf; upper surface showing the characteristic texture of *coyunda*. F. Pod; showing five stages: immature (green), intermediate green-mature stage, mature (“granada”), intermediate mature-dry stage, and dry (seed harvest). Each element is accompanied by its corresponding size scale. Photographic records obtained during agricultural seasons 1 and 2 of Porotarium Austral.

**Annex 10. Lankester plate P008 PVG - Coyunda Amarillo**. A. Seed; lateral and ventral views. B. Flower. C. Leaf; underside of the central leaflet. D. Whole leaf; upper surface showing the characteristic texture of *coyunda*. F. Pod; showing three stages: immature (green), intermediate green-mature stage, and mature (“granada”). Each element is accompanied by its corresponding size scale. Photographic records obtained during agricultural seasons 1 and 2 of Porotarium Austral.

**Annex 11. Lankester plate P009 PVG - Coyunda Kalfu**. A. Seed; lateral and ventral views. B. Flower. C. Inflorescence showing developing buds, from proximal to distal, which are white when formed and gradually turn violet. D. Whole leaf; upper surface showing the characteristic texture of *coyunda*. E. Leaf; underside of the central leaflet. F. Pod; showing five stages: immature (green), intermediate green-mature stage, mature (“granada”), intermediate mature-dry stage, and dry (seed harvest). Each element is accompanied by its corresponding size scale. Photographic records obtained during agricultural seasons 1 and 2 of Porotarium Austral.

**Annex 12. Lankester plate P010 PVG - Coyunda Blanco**. A. Seed; lateral and ventral views. B. Flower. C. Leaf; underside of the central leaflet. D. Whole leaf; upper surface showing the characteristic texture of *coyunda*. F. Pod; showing three stages: immature (green), intermediate green-mature stage, and mature (“granada”). Each element is accompanied by its corresponding size scale. Photographic records obtained during agricultural seasons 1 and 2 of Porotarium Austral.

**Annex 13. Lankester plate P012 PVG - Señorita Redondo.** A. Seed; ventral view of the two color variants, ochre or burgundy stain. B. Flower. C. Inflorescence showing the development of the flower buds, from proximal to distal. D. Leaf; showing the upper surface of the lateral leaflets and the underside of the central leaflet. E. Pod; showing five stages: immature (green), intermediate green-mature stage, mature (“granada”), intermediate mature-dry stage, and dry (seed harvest). Each element is accompanied by its corresponding size scale. Photographic records obtained during agricultural seasons 1 and 2 of Porotarium Austral.

**Annex 14. Lankester plate P013 PVM - Angelito mancha morada**. A. Seed; lateral and ventral views, note the angel-shaped stain in the ventral view. B. Flower. C. Inflorescence showing the development of the flower buds, from proximal to distal. D. Leaf; showing the upper surface. E. Pod; showing three stages: immature (green), intermediate green-mature stage, and mature (“granada”). Each element is accompanied by its corresponding size scale. Photographic records obtained during agricultural seasons 1 and 2 of Porotarium Austral.

**Annex 15. Lankester plate P015 PVM - Vaquita pintas negras**. A. Seed; lateral and ventral views. B. Flower. C. Leaf; underside of the central leaflet. D. Whole leaf; showing the upper surface. E. Pod; showing two stages: immature (green), and intermediate green-mature stage. Each element is accompanied by its corresponding size scale. Photographic records obtained during agricultural seasons 1 and 2 of Porotarium Austral.

**Annex 16. Lankester plate P016 PVG - Jardinero/Caballero**. A. Seed; lateral and ventral views. B. Flower. C. Stamens and pistil inside the flower. D. Leaf; showing the upper surface of the central and right leaflets, and the underside of the left leaflet. E. Pod; showing five stages: immature (green), intermediate green-mature stage, mature (“granada”), intermediate mature-dry stage, and dry (seed harvest). Each element is accompanied by its corresponding size scale. Photographic records obtained during agricultural seasons 1 and 2 of Porotarium Austral.

**Annex 17. Lankester plate P019 PVMG - Vaquita burdeo alargado**. A. Seed; lateral and ventral views. B. Flower. C. Leaf; showing the upper surface of the lateral leaflets and the underside of the central leaflet. D. Pod; showing five stages: immature (green), intermediate green-mature stage, mature (“granada”), intermediate mature-dry stage, and dry (seed harvest). Each element is accompanied by its corresponding size scale. Photographic records obtained during agricultural seasons 1 and 2 of Porotarium Austral.

**Annex 18. Lankester plate P026 PVG - Pitío Tricolor.** A. Seed; lateral and ventral views. B. Inflorescence and flower. C. Leaf; showing the upper surface of the lateral leaflets and the underside of the central leaflet. D. Pod; showing five stages: immature (green), intermediate green-mature stage, mature (“granada”), intermediate mature-dry stage, and dry (seed harvest). Each element is accompanied by its corresponding size scale. Photographic records obtained during agricultural seasons 1 and 2 of Porotarium Austral.

**Annex 19. Lankester plate P028 PVG - Frutilla/Araucano**. A. Seed; lateral and ventral views. B. Flower. C. Inflorescence showing the development of the buds, from proximal to distal. D. Leaf; showing the upper surface of the three leaflets. E. Pod; showing six stages: immature (green), intermediate green-mature stage, mature (“granada”), intermediate mature-dry stage, dry closed, and dry open (seed harvest). Each element is accompanied by its corresponding size scale. Photographic records obtained during agricultural seasons 1 and 2 of Porotarium Austral.

**Annex 20. Lankester plate P029 PVG - Sortelino**. A. Seed; lateral and ventral views. B. Flower. C. Pod; showing five stages: immature (green), intermediate green-mature stage, mature (“granada”), intermediate mature-dry stage, and dry (seed harvest). D. Whole leaf; showing the upper surface of the three leaflets. E. Leaf; ventral surface of the central leaflet. Each element is accompanied by its corresponding size scale. Photographic records obtained during agricultural seasons 1 and 2 of Porotarium Austral.

**Annex 21. Lankester plate P033 PVMG - Araucano**. A. Seed; lateral and ventral views. B. Flower. C. Whole leaf; showing the upper surface of the three leaflets, and the ventral surface of the central leaflet. D. Pod; showing five stages: immature (green), intermediate green-mature stage, mature (“granada”), intermediate mature-dry stage, and dry (seed harvest). Each element is accompanied by its corresponding size scale. Photographic records obtained during agricultural seasons 1 and 2 of Porotarium Austral.

**Annex 22. Lankester plate P042 PVM - Fideo**. A. Seed; lateral view. B. Flower. C. Inflorescence showing the development of the flower buds, from proximal to distal. D. Leaf; showing the upper surface of the three leaflets. E. Pod; showing three stages: immature (green), mature (“granada”), and dry open (seed harvest). Each element is accompanied by its corresponding size scale. Photographic records obtained during agricultural seasons 1 and 2 of Porotarium Austral.

**Annex 23. Lankester plate P043 PVM - Freire**. A. Seed; lateral and ventral views. B. Flower. C. Leaf; showing the upper surface of the lateral leaflets and the underside of the central leaflet, as well as the upper surface of the central leaflet. D. Pod; showing five stages: immature (green), intermediate green-mature stage, mature (“granada”), intermediate mature-dry stage, and dry open (seed harvest). Each element is accompanied by its corresponding size scale. Photographic records obtained during agricultural seasons 1 and 2 of Porotarium Austral.

**Annex 24. Lankester plate P044 PVM - Azufre .** A. Seed; lateral and ventral views. B. Flower. C. Inflorescence showing the development of the flower buds, from proximal to distal. D. Leaf; showing the upper surface of the three leaflets. E. Pod; showing three stages: immature (green), mature (“granada”), and dry (seed harvest). Each element is accompanied by its corresponding size scale. Photographic records obtained during agricultural seasons 1 and 2 of Porotarium Austral.

**Annex 25. Lankester plate P057 PVM - Sinhilo de mata**. A. Seed; lateral and ventral views. B. Flower. C. Leaf; showing the upper surface of the three leaflets. D. Pod; showing five stages: immature (green), intermediate green-mature stage, mature (“granada”), intermediate mature-dry stage, and dry (seed harvest). Each element is accompanied by its corresponding size scale. Photographic records obtained during agricultural seasons 1 and 2 of Porotarium Austral.

**Annex 26. Lankester plate P062 PVM - Capi verde amarillo.** A. Seed; lateral and ventral views. B. Flower. C. Inflorescence showing the development of the buttons, from proximal to distal. D. Leaf; showing the upper surface of the three leaflets. E. Pod; showing five stages: immature (green), intermediate green-mature stage, mature (“granada”), intermediate mature-dry stage, and dry (seed harvest). Each element is accompanied by its corresponding size scale. Photographic records obtained during agricultural seasons 1 and 2 of Porotarium Austral.
