# Supplementary material for: Colourful agrobiodiversity: morphology and phenology of bean landraces to face commodification of the commons in the southern Andes
Source: Bot Stud. 2026 Jan 15;67:1. doi: 10.1186/s40529-025-00488-6 (PMC12808010; doi:10.1186/s40529-025-00488-6)
Supplement: Supplementary file 1 — Supplementary Material 1 [file 40529_2025_488_MOESM1_ESM.zip › 40529_2025_488_MOESM1_ESM/40529_2025_488_MOESM24_ESM.docx]

|  | **PC1** | **PC2** |
| --- | --- | --- |
| Height | **0.369** | 0.072 |
| Diameter | 0.203 | 0.152 |
| Internode distance | **0.319** | 0.049 |
| Nodes before first flower | 0.098 | 0.258 |
| Central leaflet length | 0.215 | 0.226 |
| Central leaflet width | **0.376** | 0.290 |
| Petiole length | 0.214 | **0.359** |
| Petiolule length | 0.249 | -0.053 |
| Peduncle length | 0.001 | 0.167 |
| Pod length | **0.332** | -0.113 |
| Seed length | 0.145 | **-0.328** |
| Seed width | 0.241 | **-0.349** |
| Seed depth | 0.299 | **-0.296** |
| Seed volume | 0.254 | **-0.383** |
| Seed weight | 0.267 | **-0.376** |
| Eigenvalue | 8250.5 | 942.9 |
| Explained variance | 87.13% | 9.946% |
| Standard deviation | 2.17 | 2.07 |
