# Supplementary material for: Colourful agrobiodiversity: morphology and phenology of bean landraces to face commodification of the commons in the southern Andes
Source: Bot Stud. 2026 Jan 15;67:1. doi: 10.1186/s40529-025-00488-6 (PMC12808010; doi:10.1186/s40529-025-00488-6)
Supplement: Supplementary file 1 — Supplementary Material 1 [file 40529_2025_488_MOESM1_ESM.zip › 40529_2025_488_MOESM1_ESM/40529_2025_488_MOESM26_ESM.docx]

| **Landrace** | **Gardens** | **Observations** |
| --- | --- | --- |
| P1 | 6 | Late pod formation (April) |
| P6 | 7, 4, 11, 6 | Early pod formation (January) |
| P11 | 6 | Sensitive during germination stage, young leaves are susceptible to damage from rufous-tailed plantcutter (*Phytotoma rara*) |
| P15 | 1, 6 | Sensitive to frost both pre- and post-germination |
| P17 | 6 | Sensitive during germination stage (November), young leaves susceptible to damage from *P. rara* |
| P18 | 11 | Sensitive during germination stage |
| P21 | 6 | Sensitive during germination stage (November), young leaves sensitive to damage from *P. rara* |
| P25 | 6 | Sensitive during germination stage (November). |
| P29 | 6, 7, 11 | Resistant to damage from *P. rara*, late pod formation (March). |
| P30 | 6 | Young leaves sensitive to damage from *P. rara* |
| P35 | 6 | Good performance in greenhouse |
| P44 | 6, 3, 11, 8 | Sensitive post-germination (November) to frost |
| P51 | 6 | Young leaves sensitive to drought |
| P54 | 6, 11 | Sensitive post-germination (November) to heat and frost, young leaves sensitive to damage by *Phytotoma rara*. |
| P56 | 11 | Sensitive during germination stage |
| P57 | 6 | Early pod formation (January) |
| P59 | 11 | Sensitive during germination stage |
| P60 | 11 | Sensitive post-germination (November) to frost |
| P61 | 6 | Young leaves sensitive to damage from *P. rara* |
| P65 | 8, 6 | Late flowering (March) |
| P67 | 6 | Young leaves sensitive to drought and damage from *P. rara* |
| P71 | 6 | Young leaves sensitive to drought |
| P77 | 6 | Late flowering (March) |
