# Supplementary figures and images for: Colourful agrobiodiversity: morphology and phenology of bean landraces to face commodification of the commons in the southern Andes
Source: Bot Stud. 2026 Jan 15;67:1. doi: 10.1186/s40529-025-00488-6 (PMC12808010; doi:10.1186/s40529-025-00488-6)

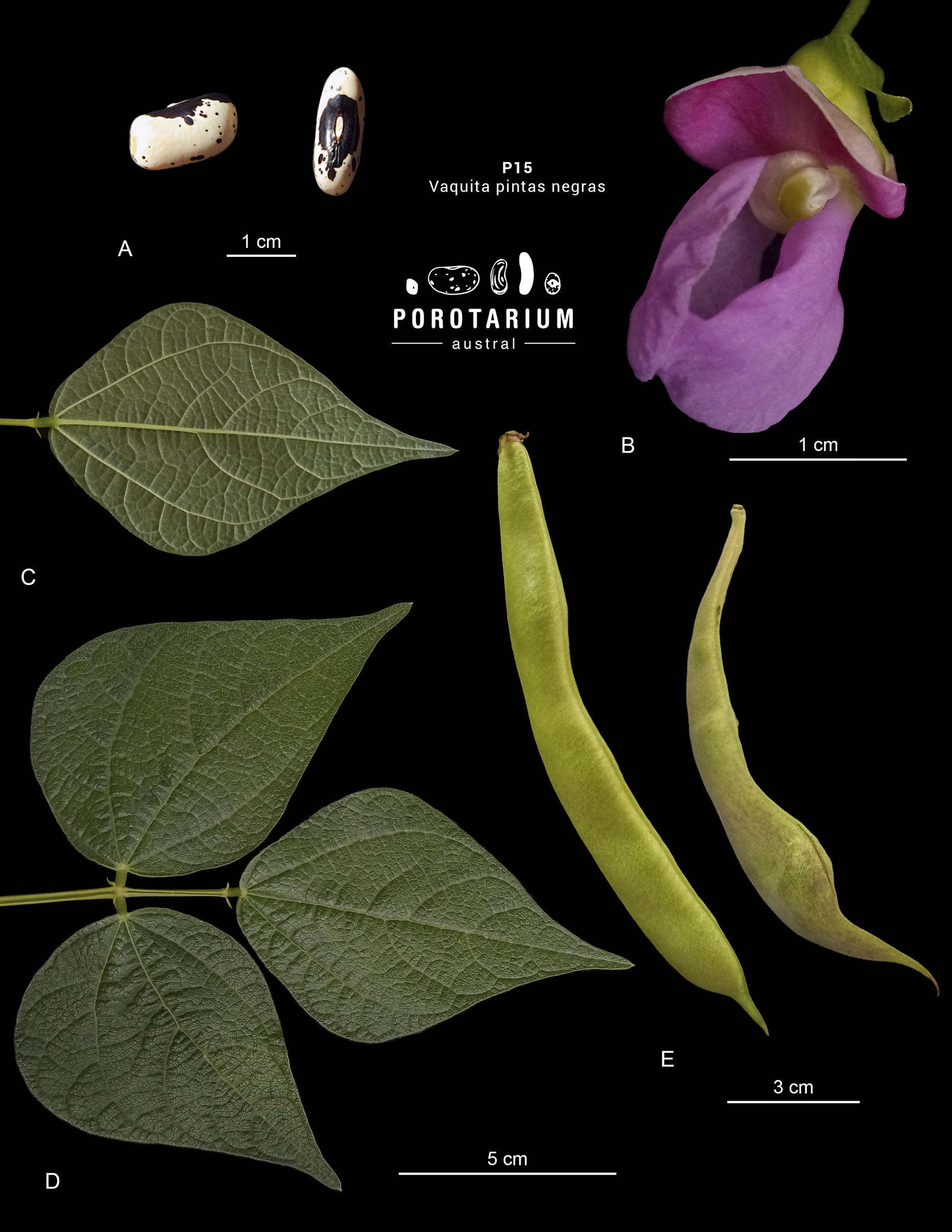

Supplement: Supplementary file 1 — Supplementary Material 1 [file 40529_2025_488_MOESM1_ESM.zip › 40529_2025_488_MOESM1_ESM/40529_2025_488_MOESM10_ESM.tif]

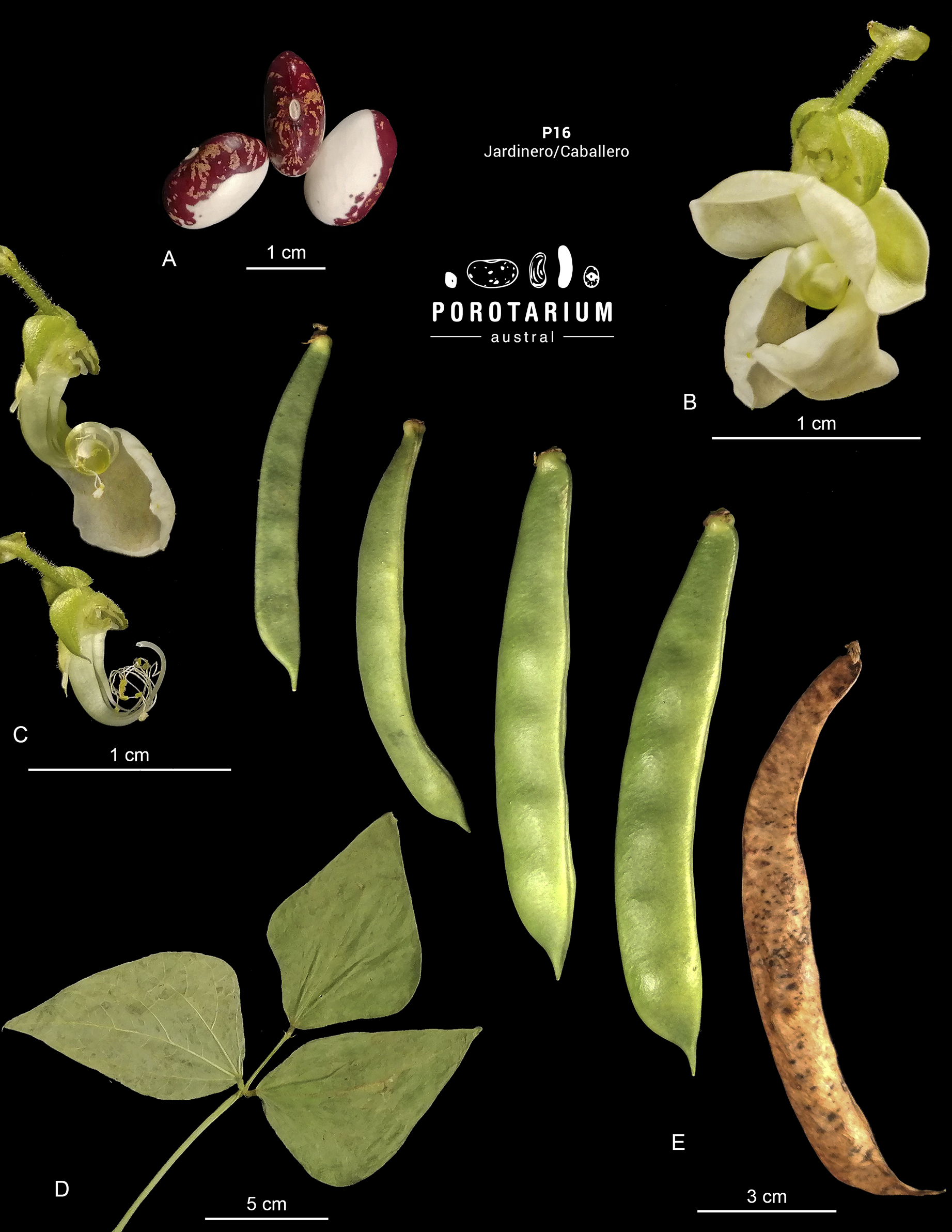

Supplement: Supplementary file 1 — Supplementary Material 1 [file 40529_2025_488_MOESM1_ESM.zip › 40529_2025_488_MOESM1_ESM/40529_2025_488_MOESM11_ESM.tif]

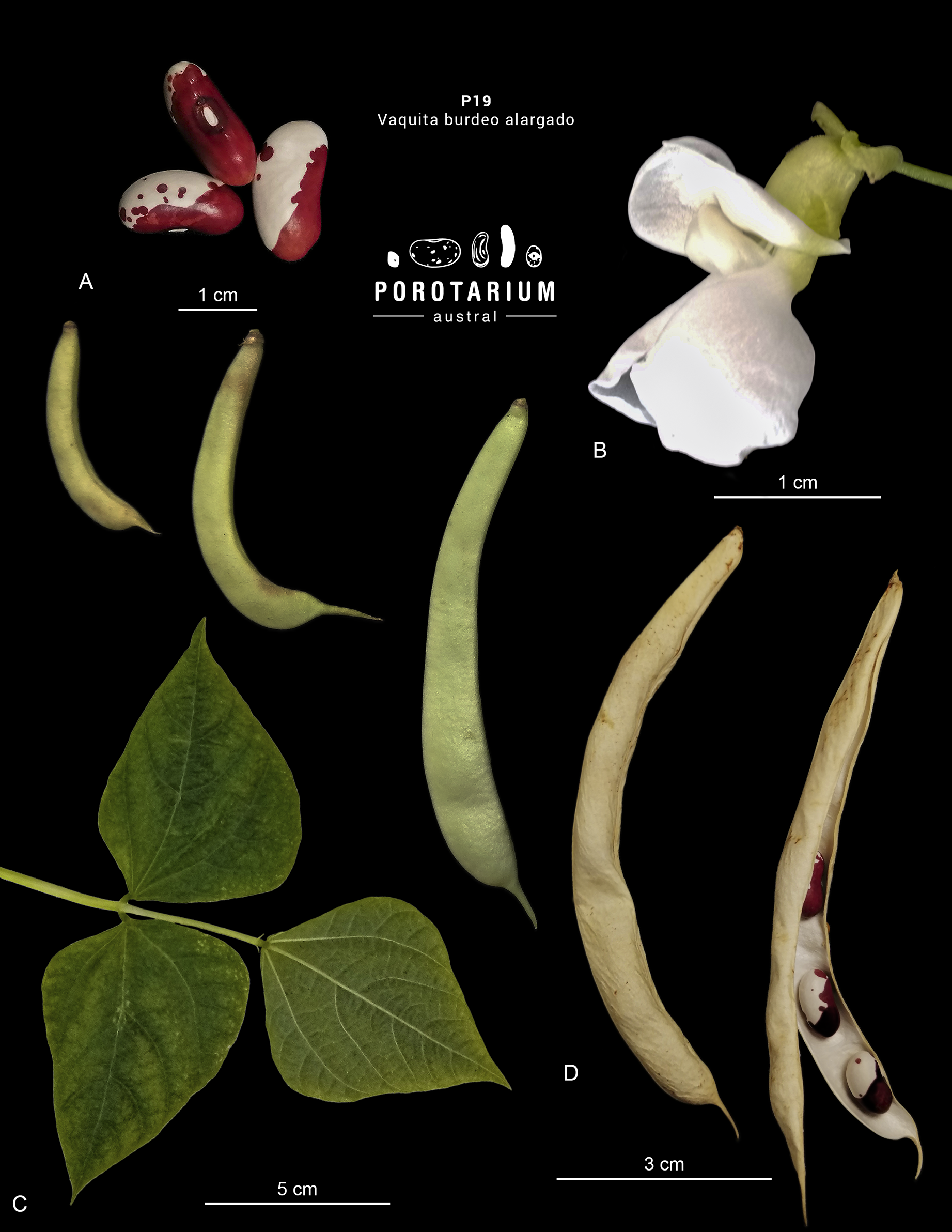

Supplement: Supplementary file 1 — Supplementary Material 1 [file 40529_2025_488_MOESM1_ESM.zip › 40529_2025_488_MOESM1_ESM/40529_2025_488_MOESM12_ESM.tif]

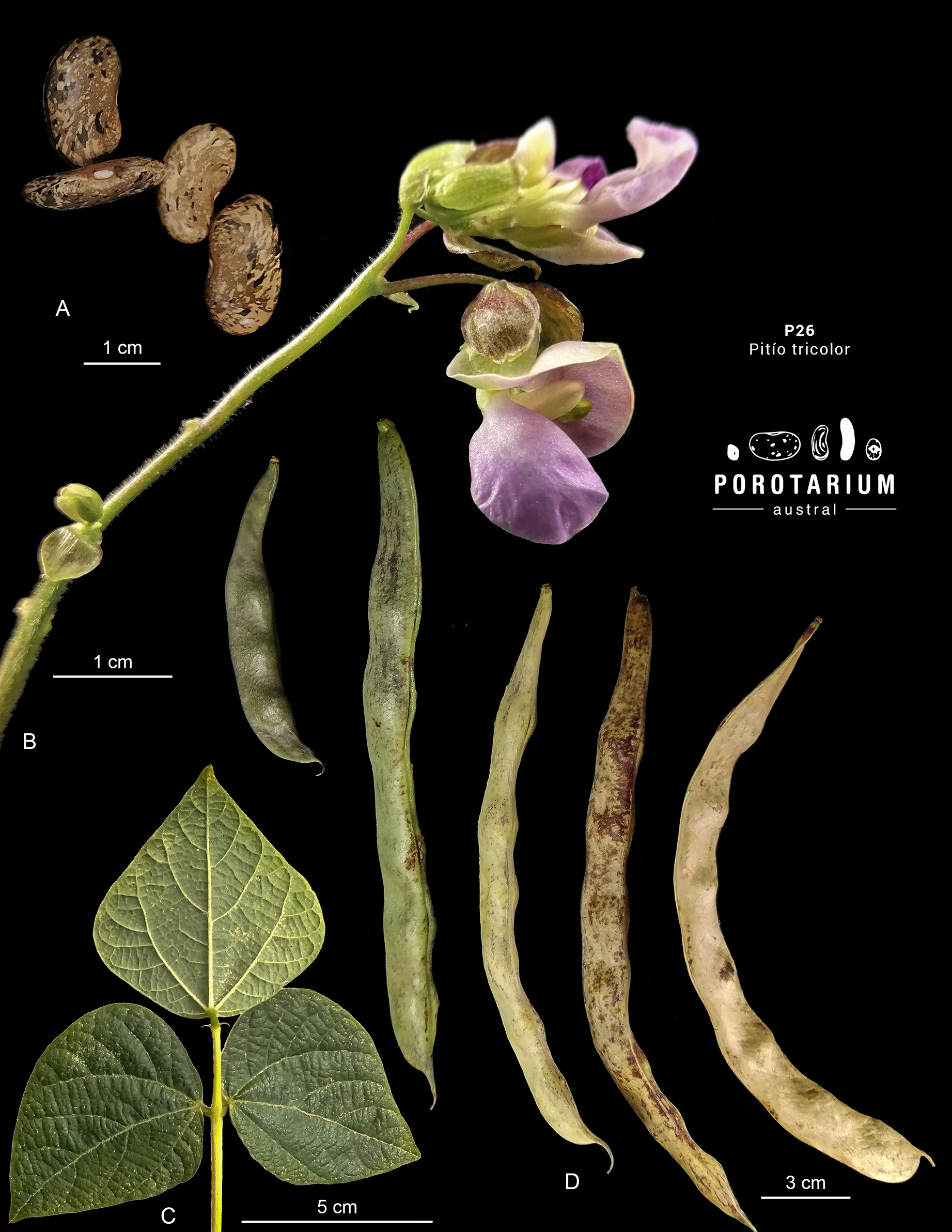

Supplement: Supplementary file 1 — Supplementary Material 1 [file 40529_2025_488_MOESM1_ESM.zip › 40529_2025_488_MOESM1_ESM/40529_2025_488_MOESM13_ESM.tif]

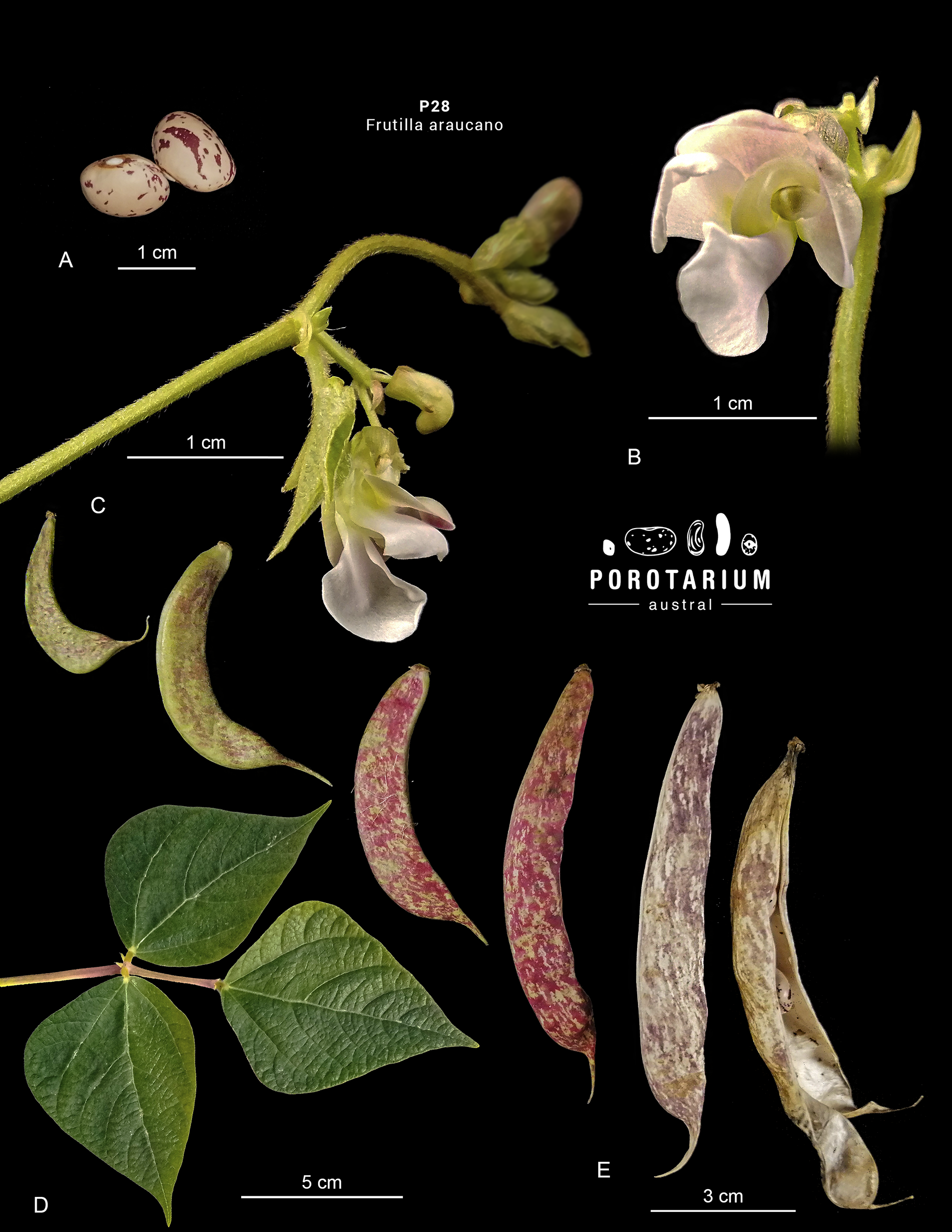

Supplement: Supplementary file 1 — Supplementary Material 1 [file 40529_2025_488_MOESM1_ESM.zip › 40529_2025_488_MOESM1_ESM/40529_2025_488_MOESM14_ESM.tif]

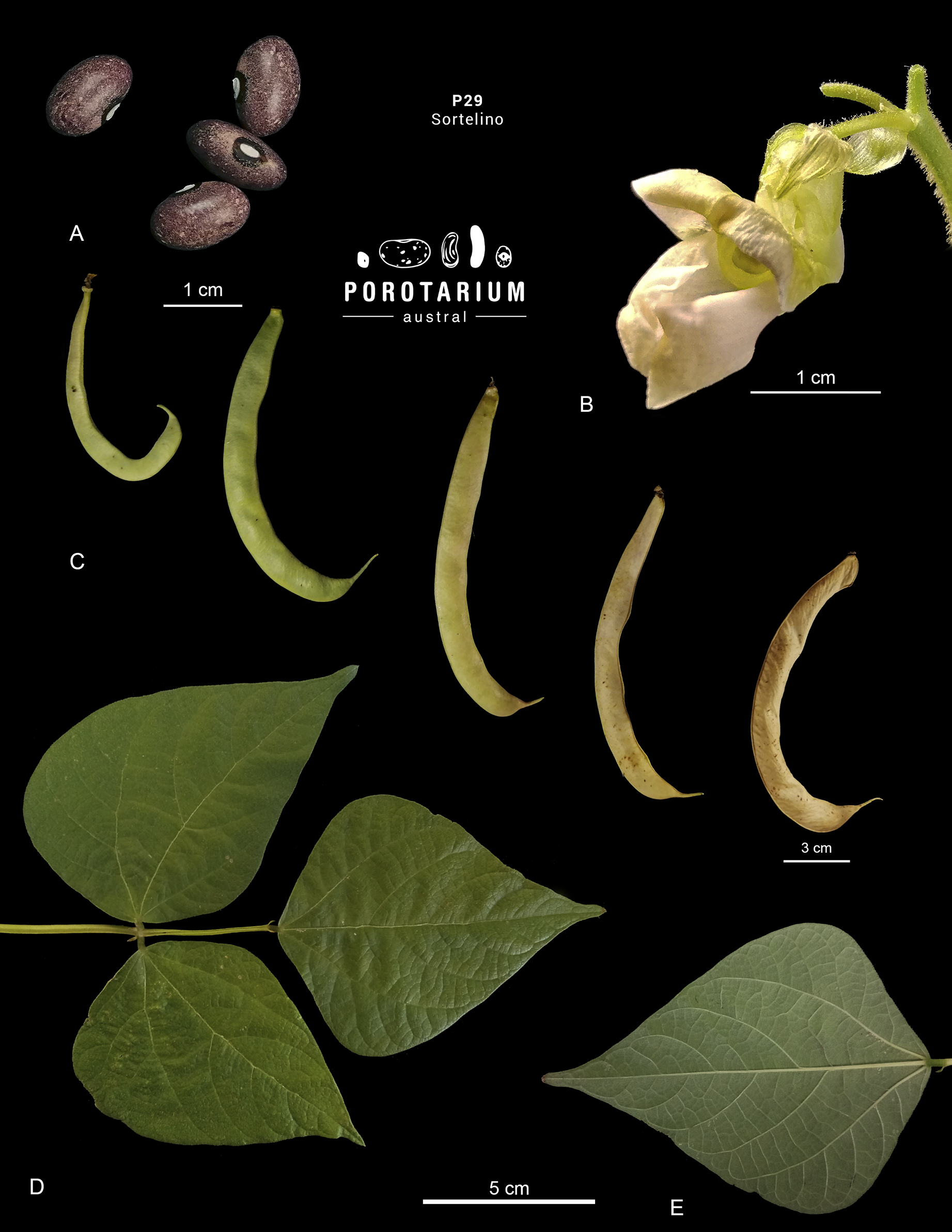

Supplement: Supplementary file 1 — Supplementary Material 1 [file 40529_2025_488_MOESM1_ESM.zip › 40529_2025_488_MOESM1_ESM/40529_2025_488_MOESM15_ESM.tif]

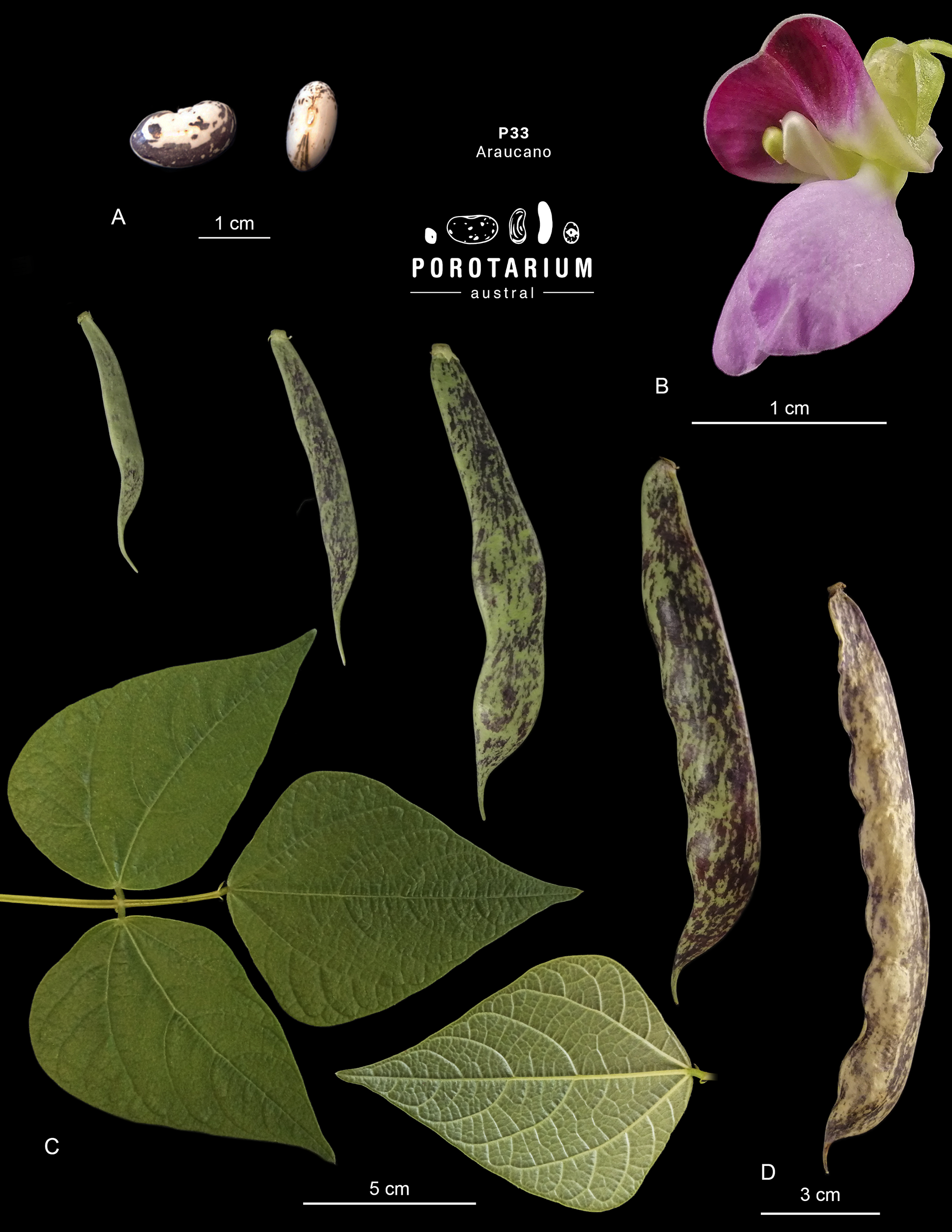

Supplement: Supplementary file 1 — Supplementary Material 1 [file 40529_2025_488_MOESM1_ESM.zip › 40529_2025_488_MOESM1_ESM/40529_2025_488_MOESM16_ESM.tif]

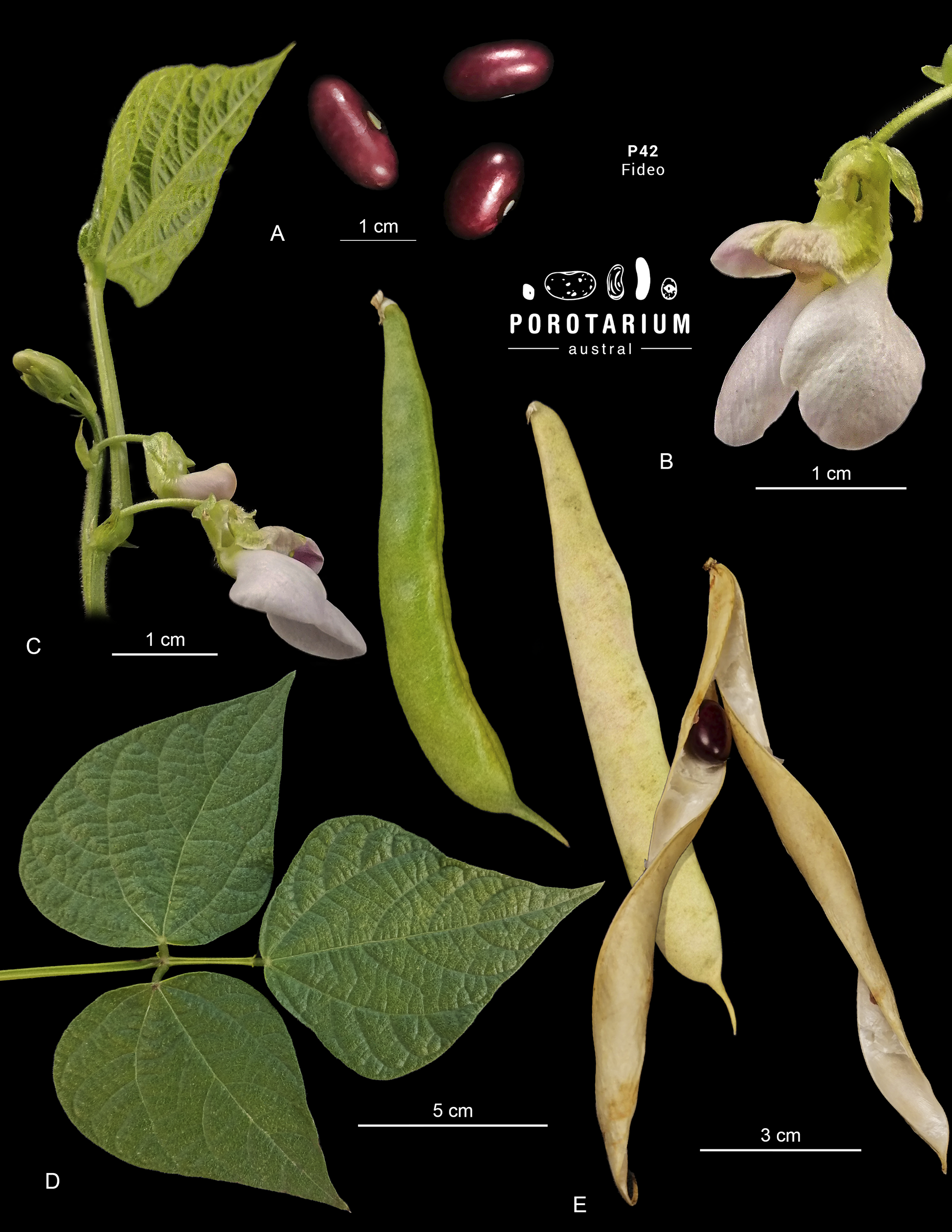

Supplement: Supplementary file 1 — Supplementary Material 1 [file 40529_2025_488_MOESM1_ESM.zip › 40529_2025_488_MOESM1_ESM/40529_2025_488_MOESM17_ESM.tif]

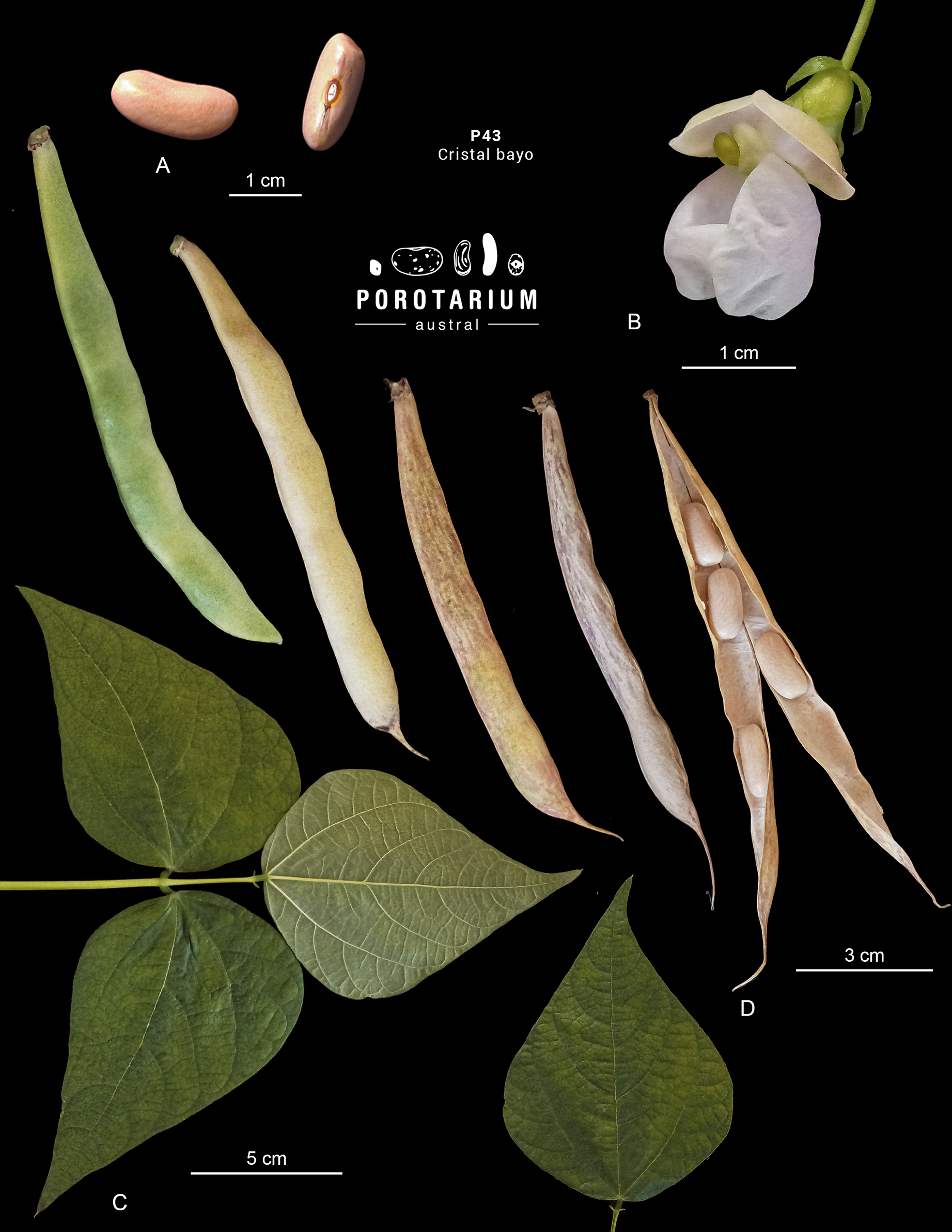

Supplement: Supplementary file 1 — Supplementary Material 1 [file 40529_2025_488_MOESM1_ESM.zip › 40529_2025_488_MOESM1_ESM/40529_2025_488_MOESM18_ESM.tif]

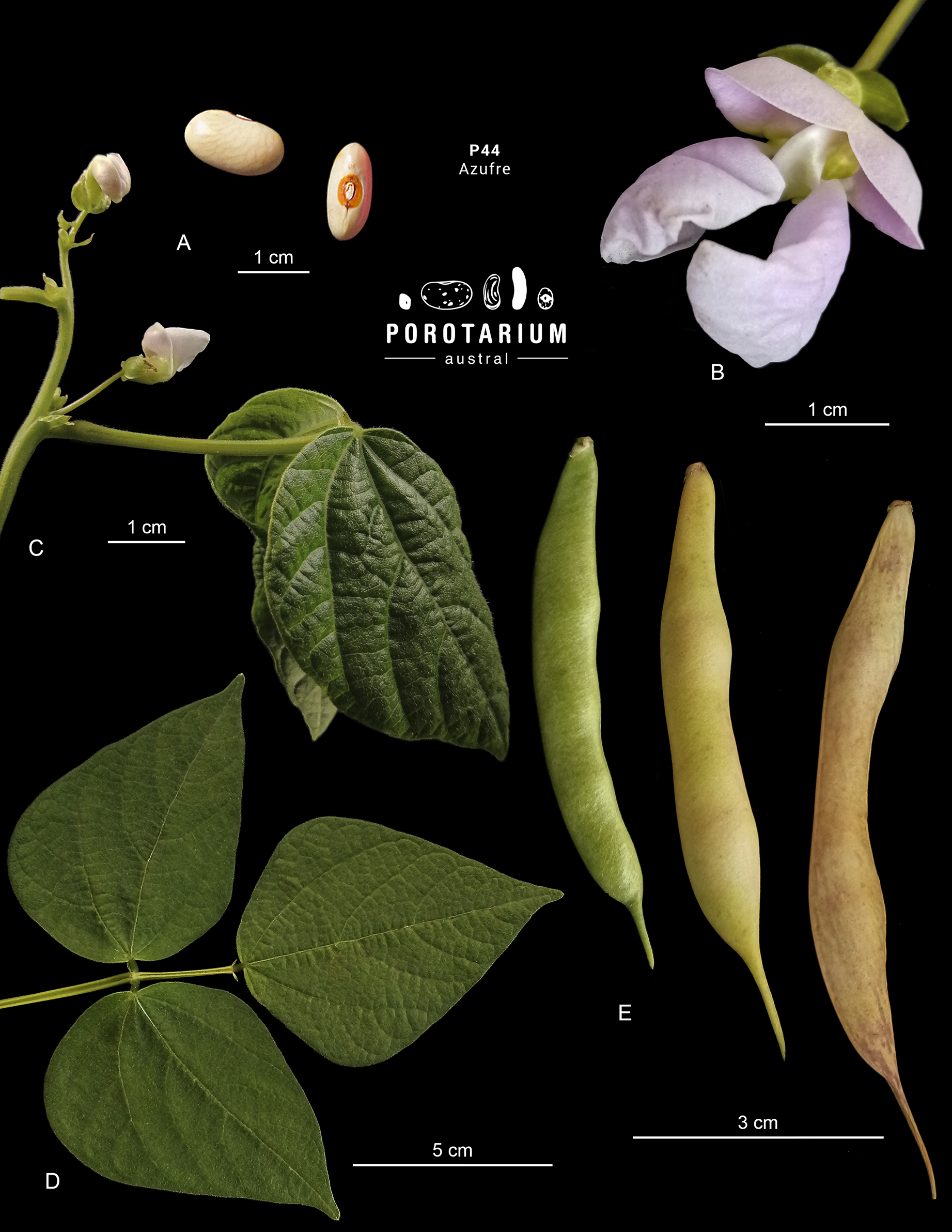

Supplement: Supplementary file 1 — Supplementary Material 1 [file 40529_2025_488_MOESM1_ESM.zip › 40529_2025_488_MOESM1_ESM/40529_2025_488_MOESM19_ESM.tif]

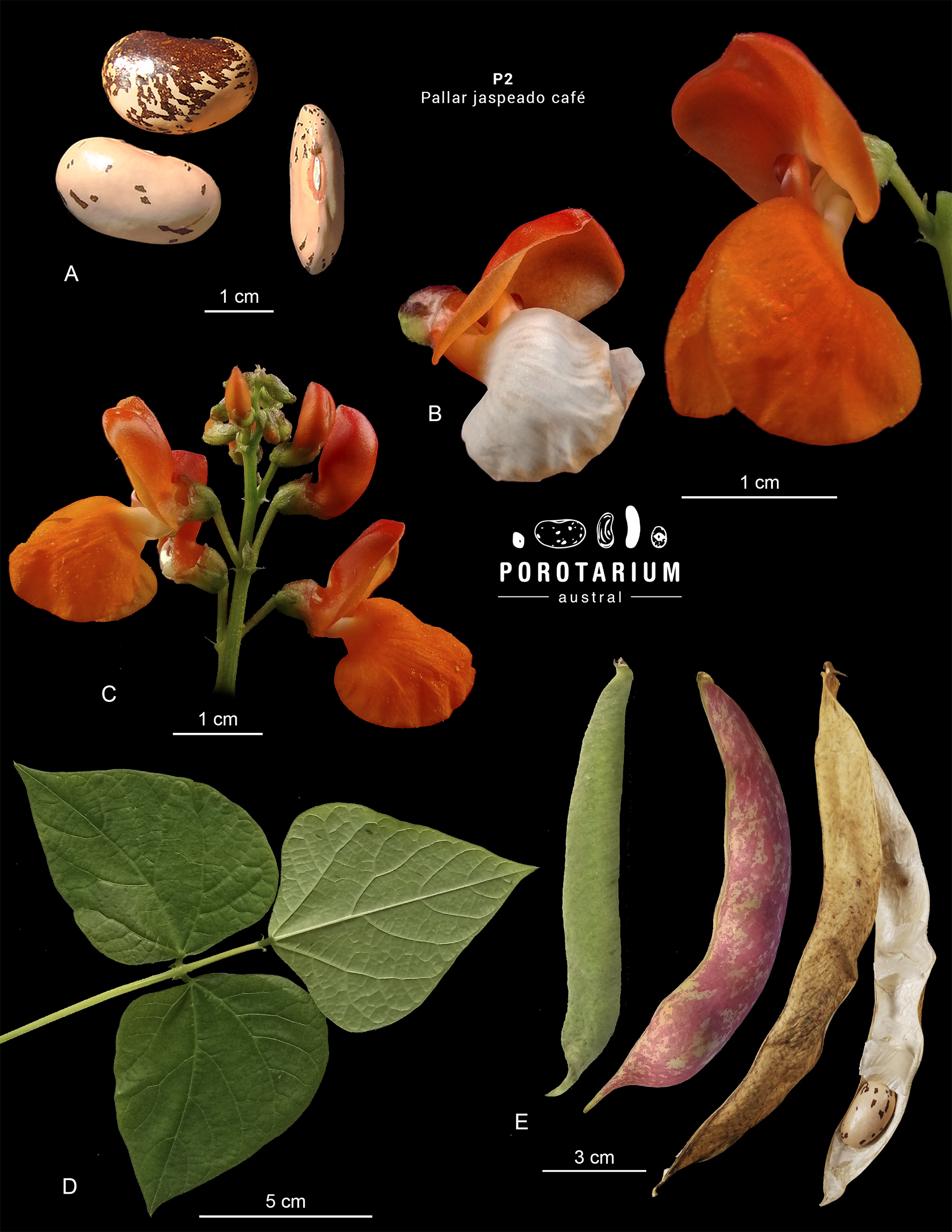

Supplement: Supplementary file 1 — Supplementary Material 1 [file 40529_2025_488_MOESM1_ESM.zip › 40529_2025_488_MOESM1_ESM/40529_2025_488_MOESM1_ESM.tif]

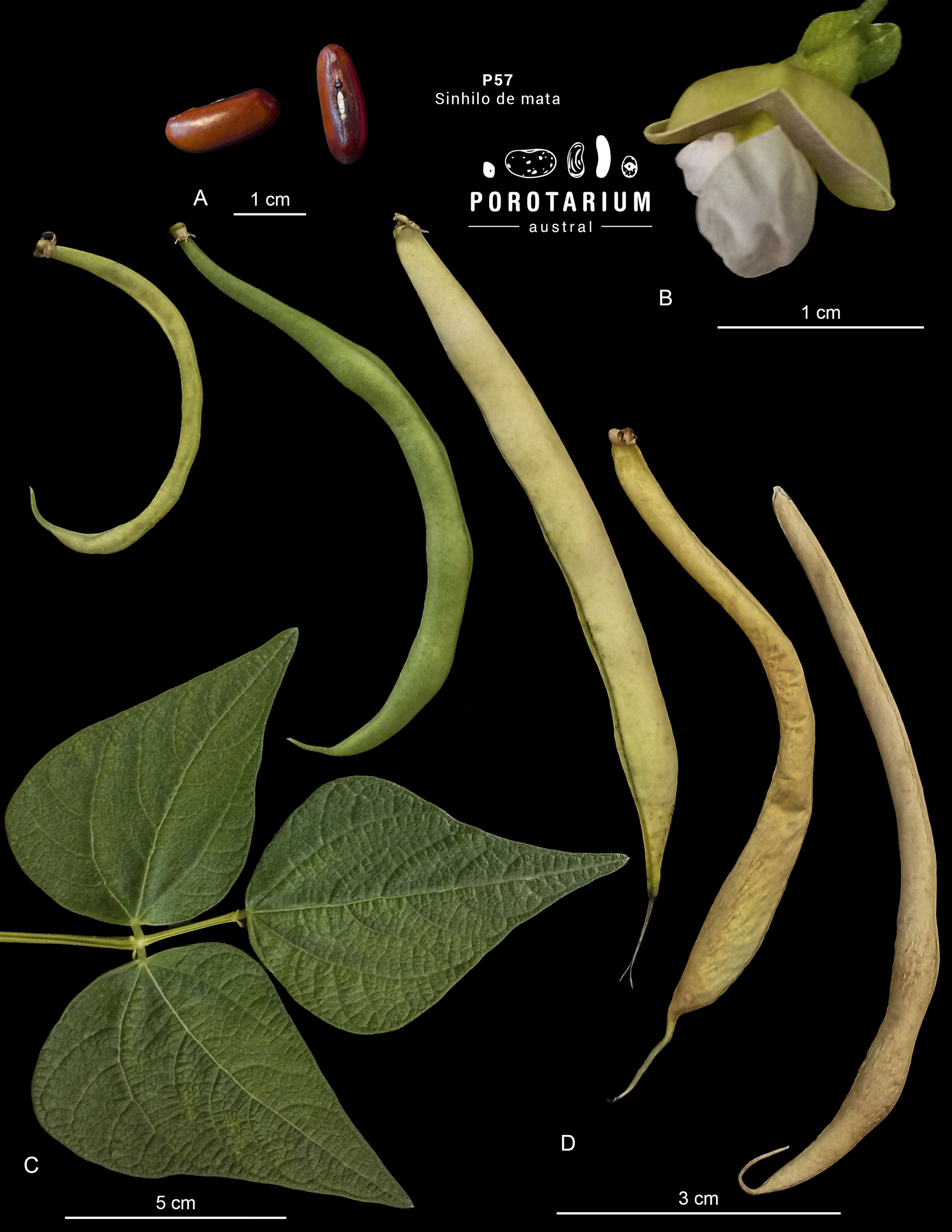

Supplement: Supplementary file 1 — Supplementary Material 1 [file 40529_2025_488_MOESM1_ESM.zip › 40529_2025_488_MOESM1_ESM/40529_2025_488_MOESM20_ESM.tif]

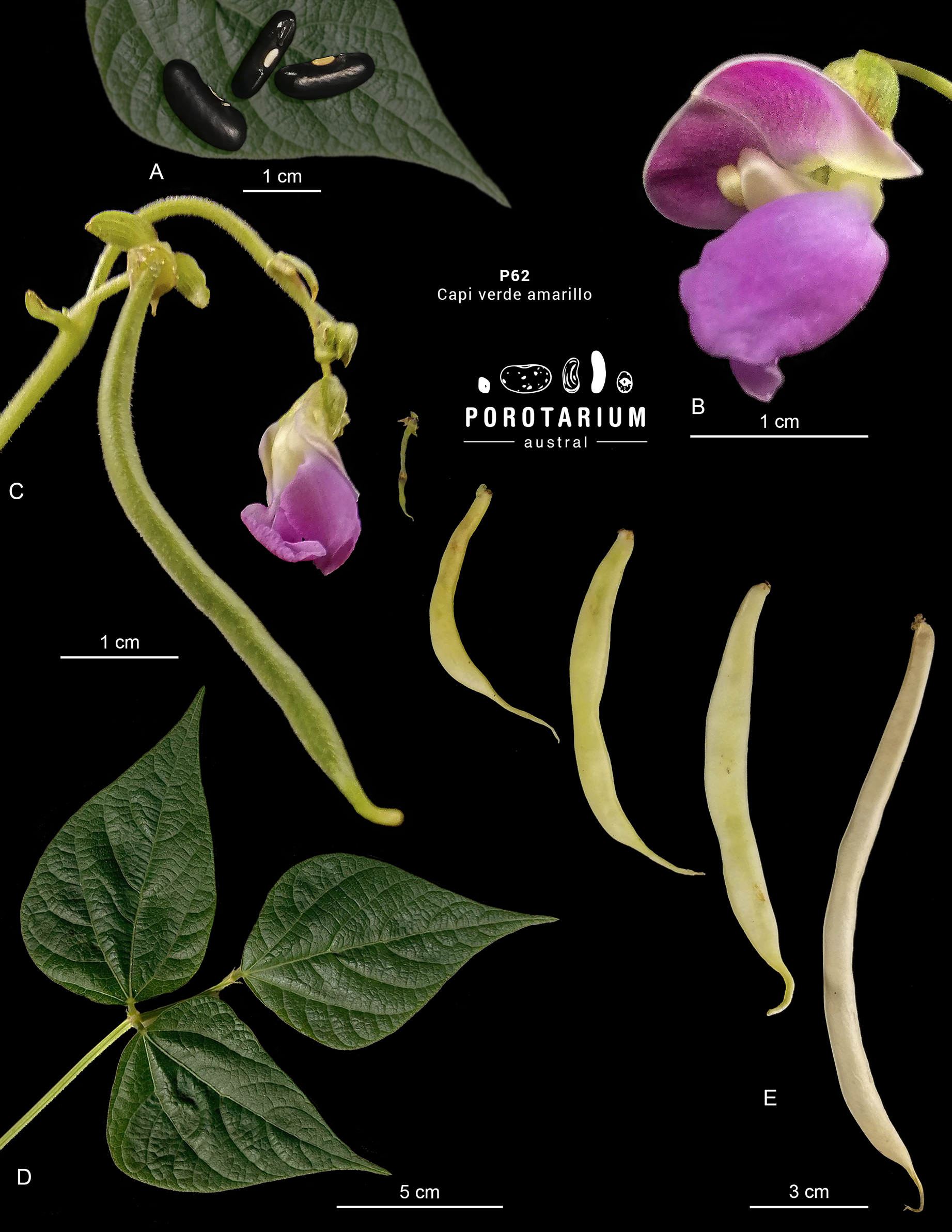

Supplement: Supplementary file 1 — Supplementary Material 1 [file 40529_2025_488_MOESM1_ESM.zip › 40529_2025_488_MOESM1_ESM/40529_2025_488_MOESM21_ESM.tif]

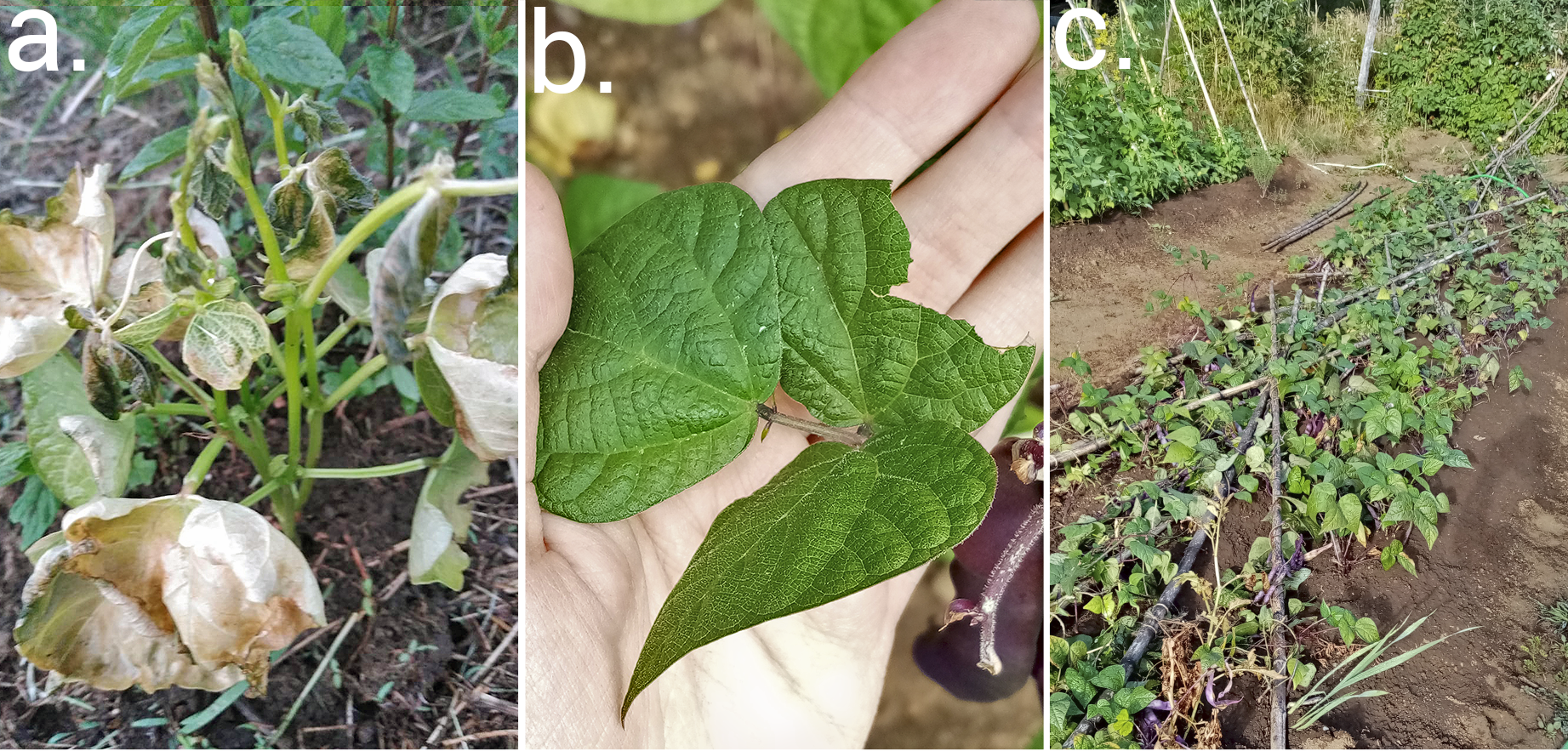

Supplement: Supplementary file 1 — Supplementary Material 1 [file 40529_2025_488_MOESM1_ESM.zip › 40529_2025_488_MOESM1_ESM/40529_2025_488_MOESM25_ESM.tif]

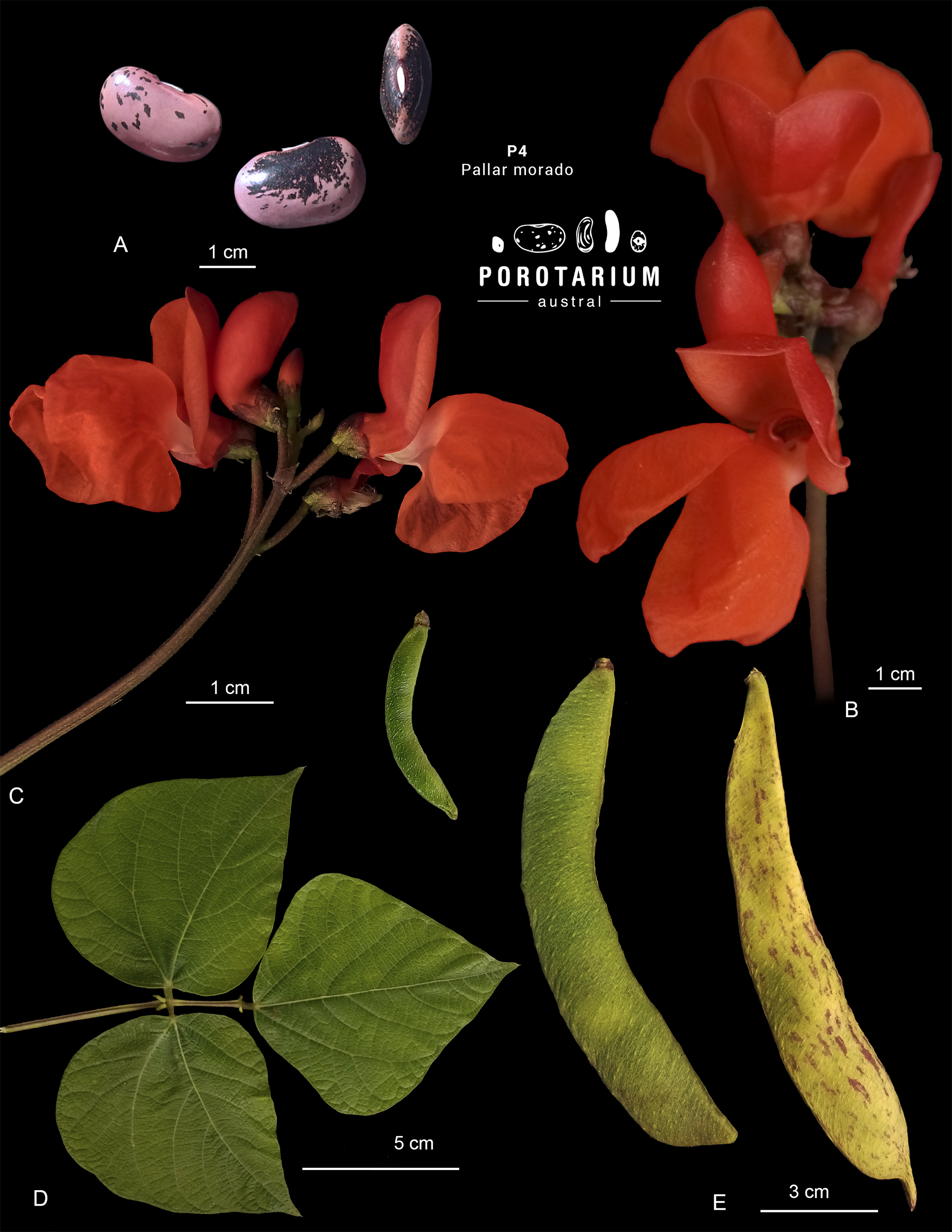

Supplement: Supplementary file 1 — Supplementary Material 1 [file 40529_2025_488_MOESM1_ESM.zip › 40529_2025_488_MOESM1_ESM/40529_2025_488_MOESM2_ESM.tif]

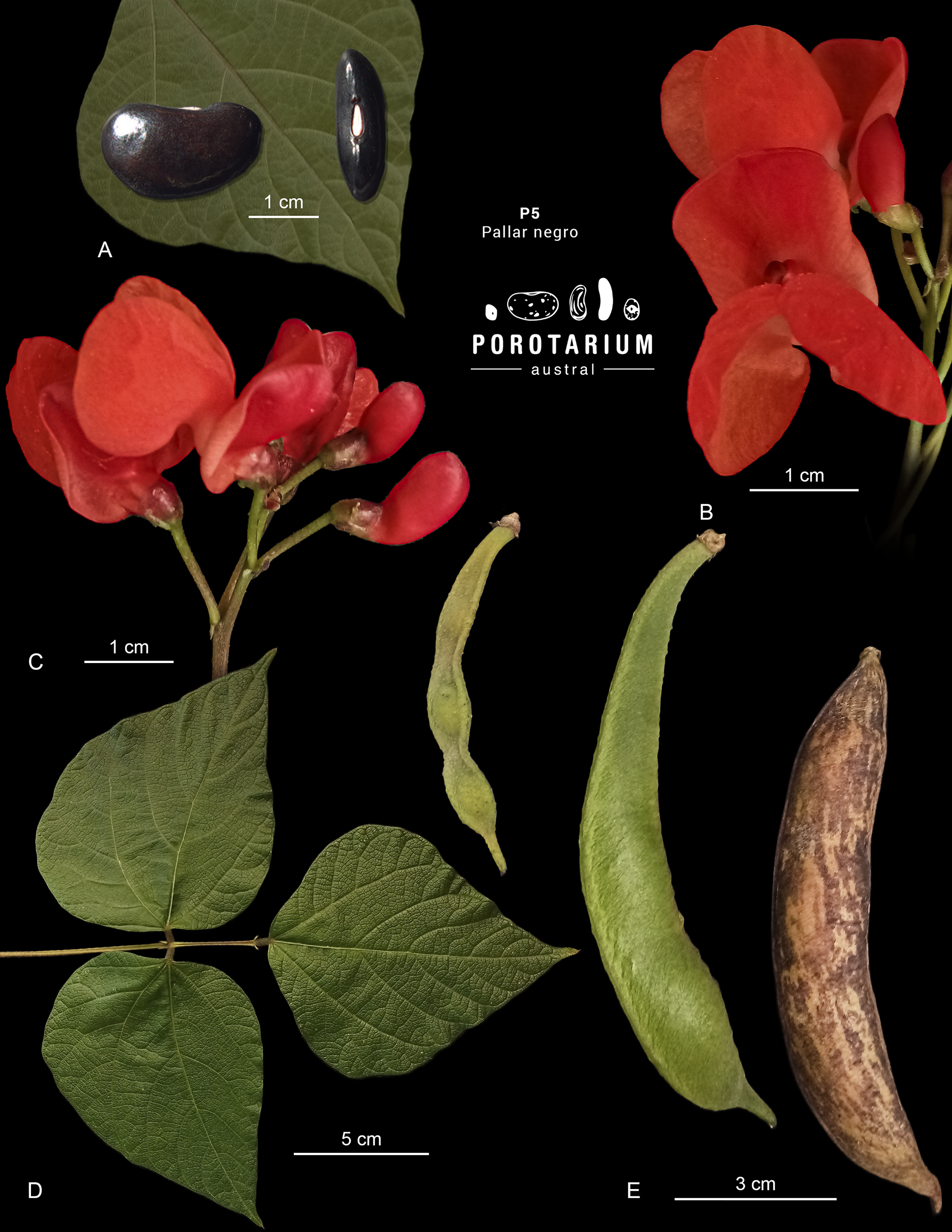

Supplement: Supplementary file 1 — Supplementary Material 1 [file 40529_2025_488_MOESM1_ESM.zip › 40529_2025_488_MOESM1_ESM/40529_2025_488_MOESM3_ESM.tif]

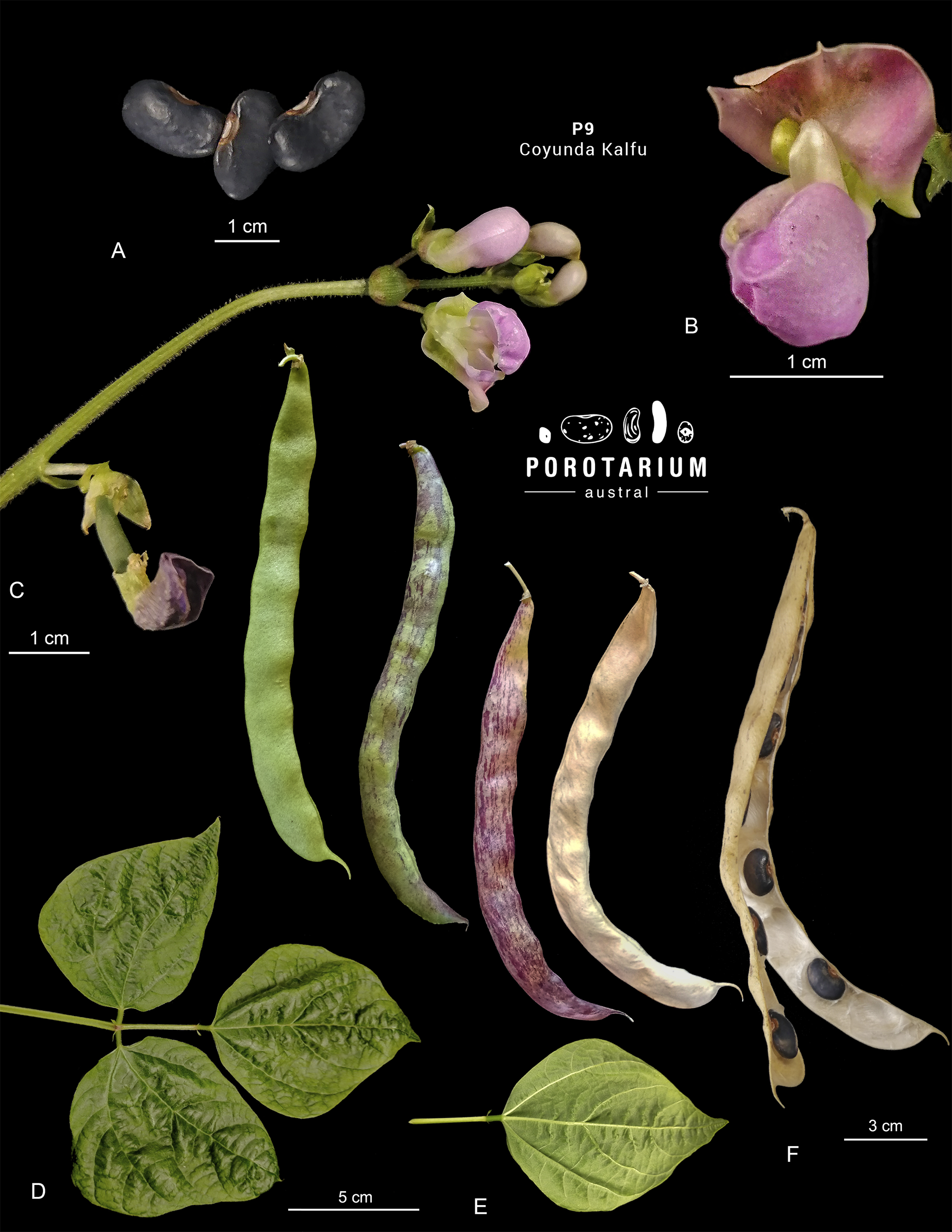

Supplement: Supplementary file 1 — Supplementary Material 1 [file 40529_2025_488_MOESM1_ESM.zip › 40529_2025_488_MOESM1_ESM/40529_2025_488_MOESM4_ESM.tif]

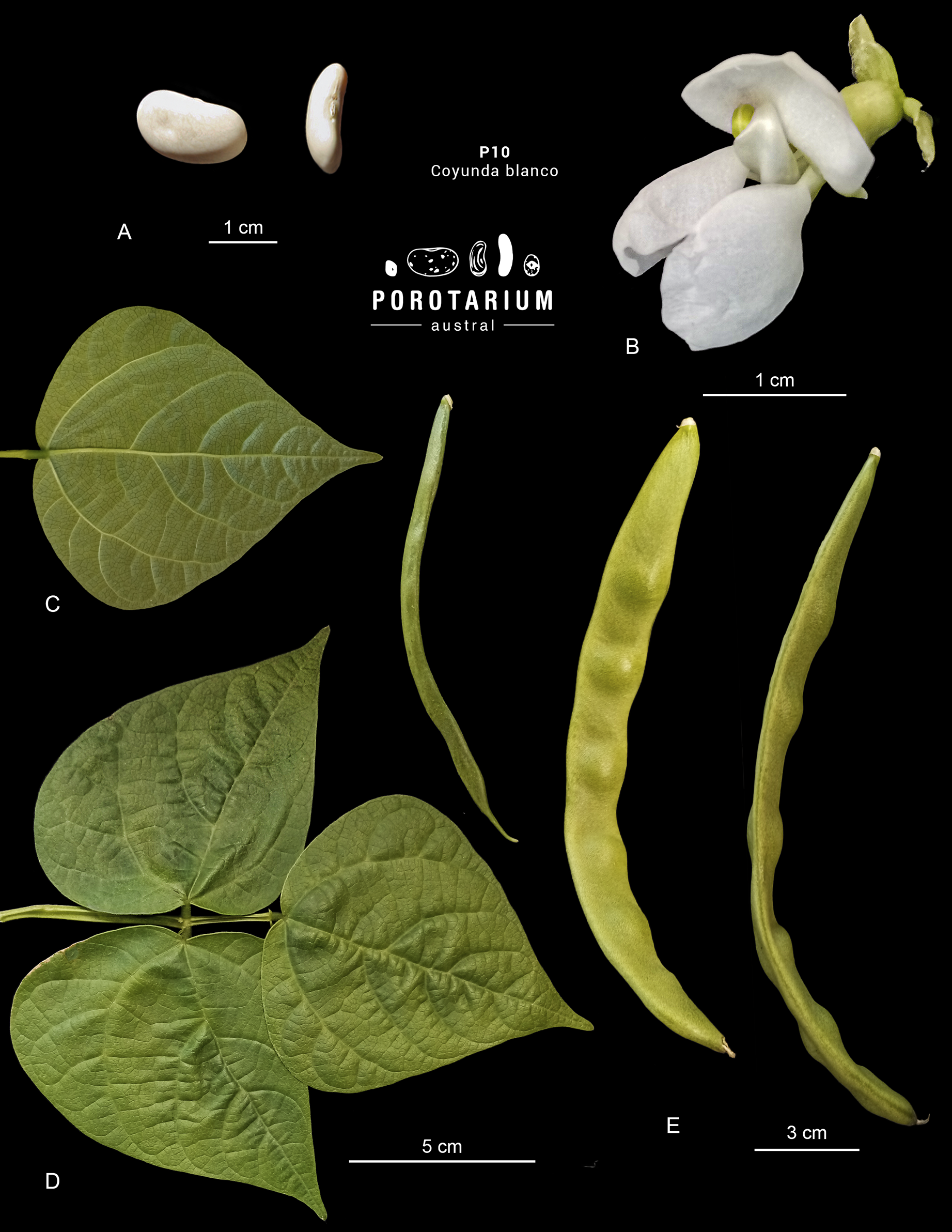

Supplement: Supplementary file 1 — Supplementary Material 1 [file 40529_2025_488_MOESM1_ESM.zip › 40529_2025_488_MOESM1_ESM/40529_2025_488_MOESM5_ESM.tif]

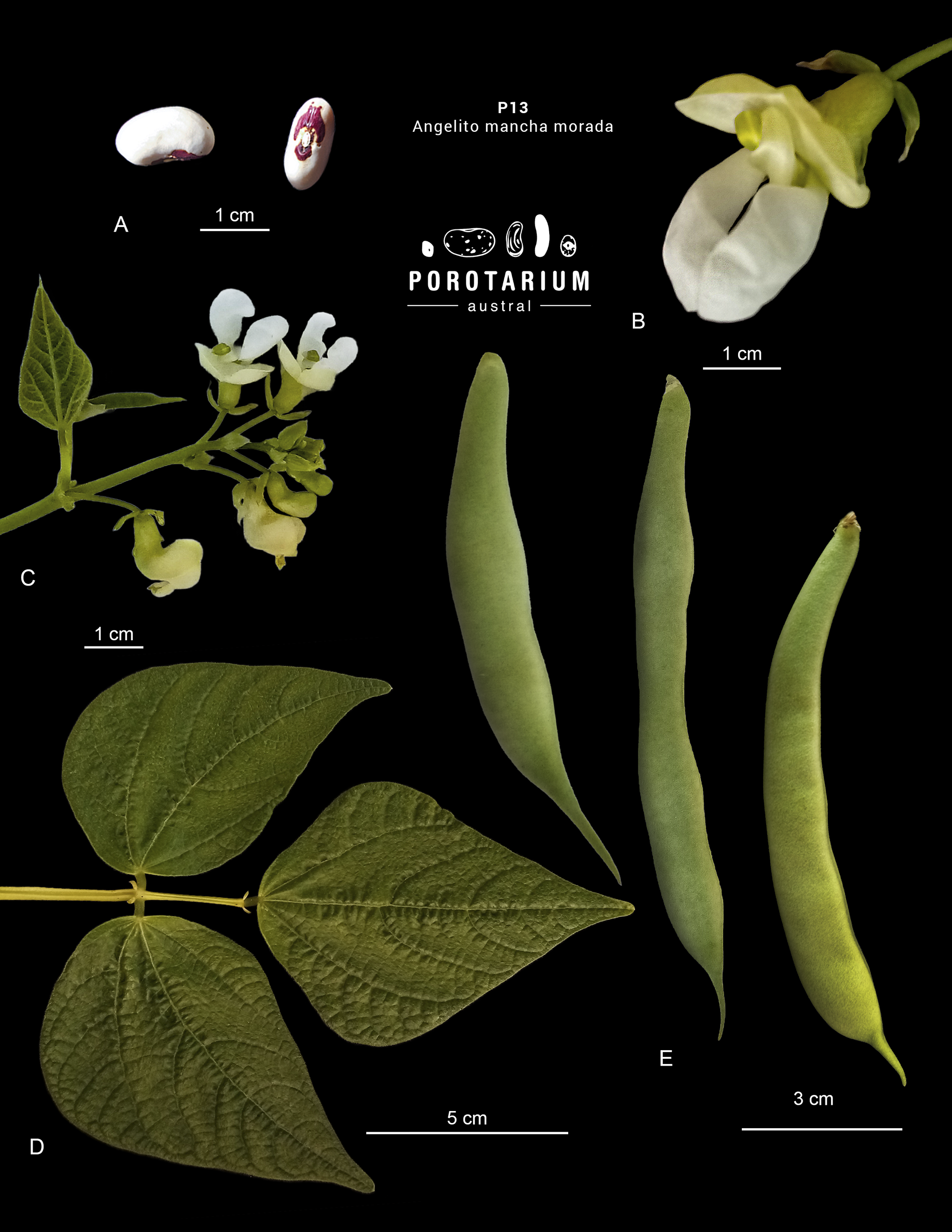

Supplement: Supplementary file 1 — Supplementary Material 1 [file 40529_2025_488_MOESM1_ESM.zip › 40529_2025_488_MOESM1_ESM/40529_2025_488_MOESM6_ESM.tif]

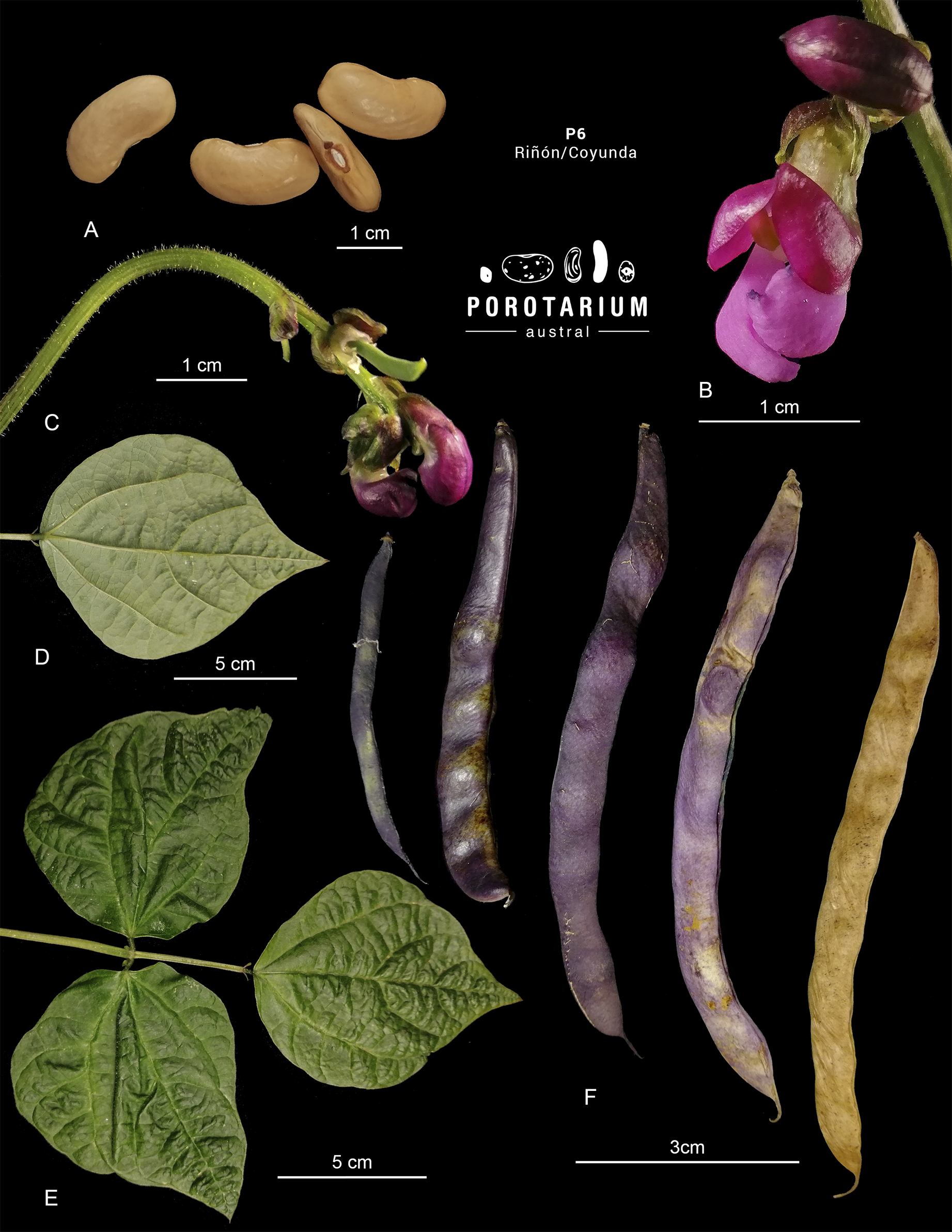

Supplement: Supplementary file 1 — Supplementary Material 1 [file 40529_2025_488_MOESM1_ESM.zip › 40529_2025_488_MOESM1_ESM/40529_2025_488_MOESM7_ESM.tif]

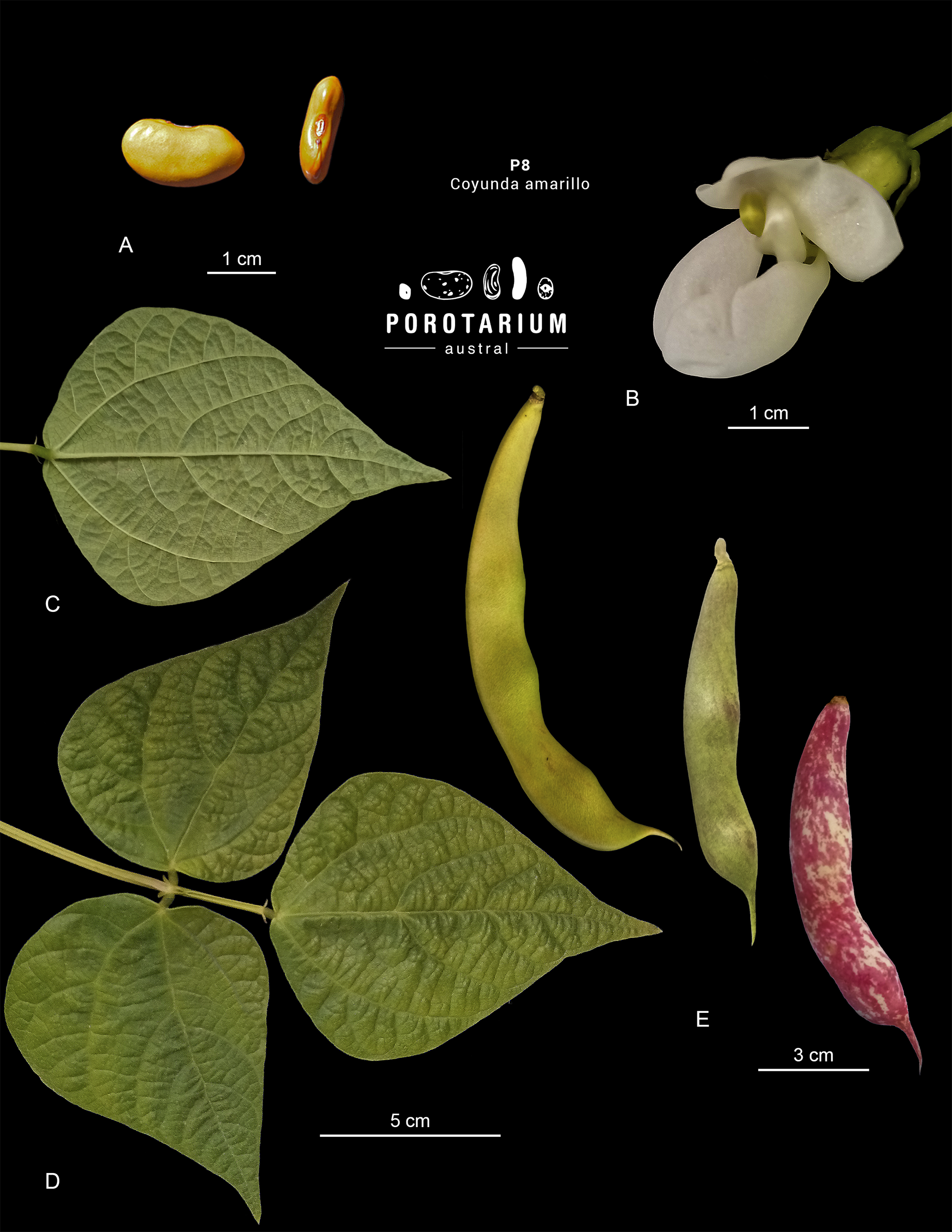

Supplement: Supplementary file 1 — Supplementary Material 1 [file 40529_2025_488_MOESM1_ESM.zip › 40529_2025_488_MOESM1_ESM/40529_2025_488_MOESM8_ESM.tif]

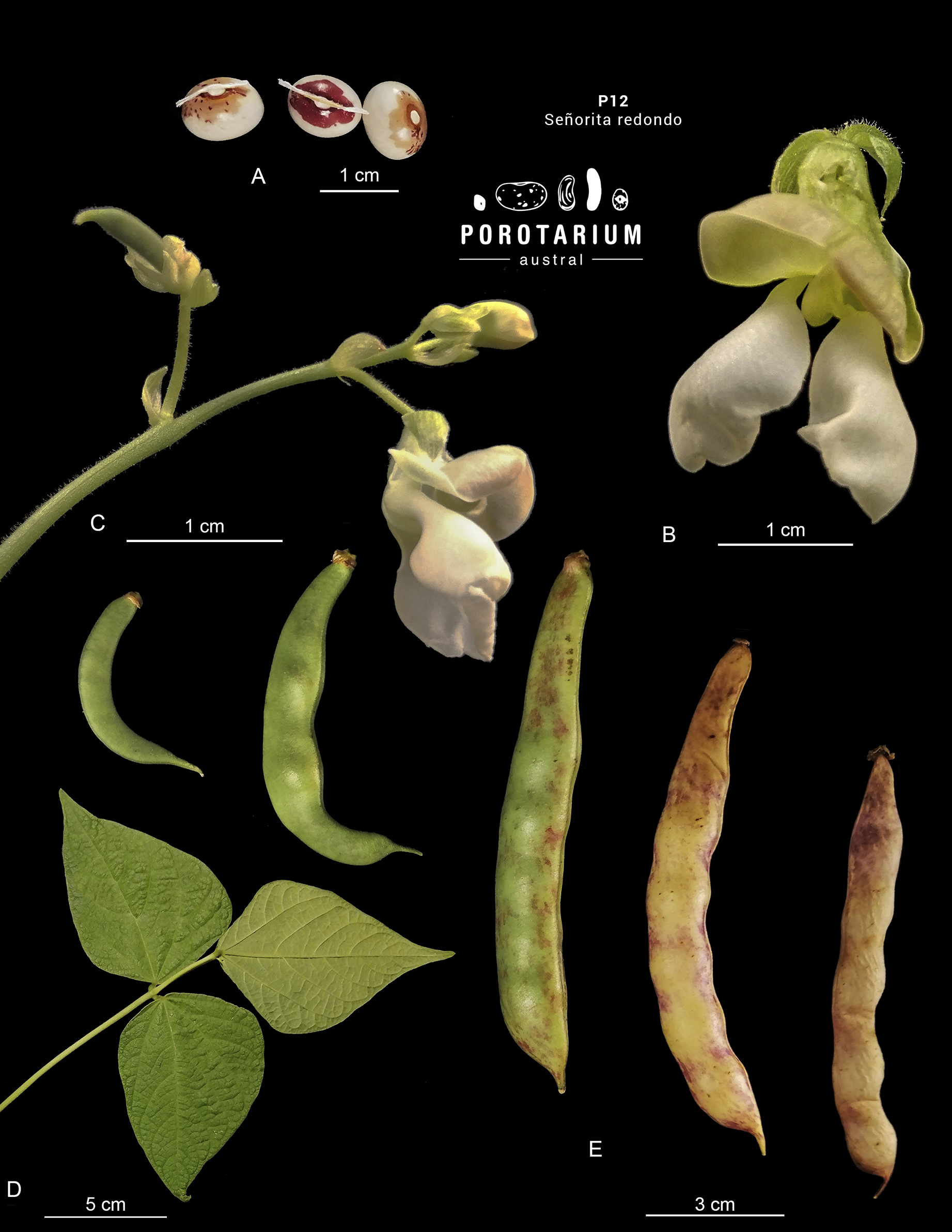

Supplement: Supplementary file 1 — Supplementary Material 1 [file 40529_2025_488_MOESM1_ESM.zip › 40529_2025_488_MOESM1_ESM/40529_2025_488_MOESM9_ESM.tif]
